# Comparisons within the Rice GA 2-Oxidase Gene Family Revealed Three Dominant Paralogs and a Functional Attenuated Gene that Led to the Identification of Four Amino Acid Variants Associated with GA Deactivation Capability

**DOI:** 10.1186/s12284-021-00499-4

**Published:** 2021-07-28

**Authors:** Kun-Ting Hsieh, Yi-Ting Chen, Ting-Jen Hu, Shih-Min Lin, Chih-Hung Hsieh, Su-Hui Liu, Shiau-Yu Shiue, Shuen-Fang Lo, I-Wen Wang, Ching-Shan Tseng, Liang-Jwu Chen

**Affiliations:** 1 Institute of Molecular Biology, National Chung Hsing University, Taichung, 40227 Taiwan; 2 Biotechnology Center, National Chung Hsing University, Taichung, 40227 Taiwan; 3 Division of Biotechnology, Taiwan Agriculture Research Institute, Taichung, 41362 Taiwan

**Keywords:** Duplicate genes, Expression profile, GA 2-oxidase gene family, GA deactivation capability, Phylogenetic footprinting, Regulatory hypofunctionalization, Sequence divergence

## Abstract

**Background:**

GA 2-oxidases (GA2oxs) are involved in regulating GA homeostasis in plants by inactivating bioactive GAs through 2β-hydroxylation. Rice GA2oxs are encoded by a family of 10 genes; some of them have been characterized, but no comprehensive comparisons for all these genes have been conducted.

**Results:**

Rice plants with nine functional GA2oxs were demonstrated in the present study, and these genes not only were differentially expressed but also revealed various capabilities for GA deactivation based on their height-reducing effects in transgenic plants. Compared to that of wild-type plants, the relative plant height (RPH) of transgenic plants was scored to estimate their reducing effects, and 8.3% to 59.5% RPH was observed. Phylogenetic analysis of class I GA2ox genes revealed two functionally distinct clades in the Poaceae. The *OsGA2ox3*, *4*, and *8* genes belonging to clade A showed the most severe effect (8.3% to 8.7% RPH) on plant height reduction, whereas the *OsGA2ox7* gene belonging to clade B showed the least severe effect (59.5% RPH). The clade A *OsGA2ox3* gene contained two conserved C186/C194 amino acids that were crucial for enzymatic activity. In the present study, these amino acids were replaced with *OsGA2ox7*-conserved arginine (C186R) and proline (C194P), respectively, or simultaneously (C186R/C194P) to demonstrate their importance *in planta*. Another two amino acids, Q220 and Y274, conserved in *OsGA2ox3* were substituted with glutamic acid (E) and phenylalanine (F), respectively, or simultaneously to show their significance *in planta*. In addition, through sequence divergence, RNA expression profile and GA deactivation capability analyses, we proposed that *OsGA2ox1*, *OsGA2ox3* and *OsGA2ox6* function as the predominant paralogs in each of their respective classes.

**Conclusions:**

This study demonstrates rice has nine functional GA2oxs and the class I GA2ox genes are divided into two functionally distinct clades. Among them, the *OsGA2ox7* of clade B is a functional attenuated gene and the *OsGA2ox1*, *OsGA2ox3* and *OsGA2ox6* are the three predominant paralogs in the family.

**Supplementary Information:**

The online version contains supplementary material available at 10.1186/s12284-021-00499-4.

## Background

The phytohormone gibberellin (GA) regulates a broad spectrum of plant growth and development aspects, including seed development, germination, stem elongation, leaf expansion, flowering and fruit development (Yamaguchi 2008). Although more than 130 GAs have been identified (MacMillan 2001), only GA_1_, GA_3_, GA_4_ and GA_7_ are recognized as major bioactive GAs in plants. The availability of bioactive GAs in plant tissues must be homeostatically maintained for proper plant growth, which is precisely regulated by both GA biosynthetic and catabolic enzymes.

The late stage of GA biosynthesis and GA catabolism in the cytosol are governed by three types of GA oxidases in the 2-oxoglutarate-dependent dioxygenase (2OGD) enzyme family (Kawai et al. 2014). The GA 20-oxidase (GA20ox), GA 3-oxidase (GA3ox) and GA 2-oxidase (GA2ox) members of the 2OGD family are thought to be the main enzymes involved in regulating GA homeostasis in plants during growth and in response to environmental signals (Yamaguchi 2008). Among them, GA20oxs and GA3oxs are GA biosynthetic enzymes (Toyomasu et al. 1997; Itoh et al. 2001), and GA2oxs are recognized as GA deactivation enzymes that convert GA precursors or bioactive GAs into inactive GAs by 2β-hydroxylation (Thomas et al. 1999). GA2ox is prevalent in seed plant species and encoded by gene family: for instance, nine in Arabidopsis (Lange and Lange 2020), ten in rice (Lo et al. 2008), eleven in *Brachypodium distachyon* (Pearce et al. 2015), eight in tomato (Chen et al. 2016) and 14 in *Medicago truncatula* (Kim et al. 2019) have been previously reported. Phylogenetic analysis based on protein sequences classified GA2oxs into three classes (Additional file: Fig. S1, Lee and Zeevaart 2005): class I and class II, whose members catalyze C_19_-GAs, are referred to as C_19_-type GA2oxs, and class III, whose members catalyze C_20_-GAs, are referred to as C_20_-type GA2oxs (Schomburg et al. 2003; Lo et al. 2008).

To explore how GA2ox can form a large gene family and how each gene evolved, numerous evolutionary analyses have been performed in land plant lineages (Han and Zhu 2011; Kawai et al. 2014; Huang et al. 2015; Takehara et al. 2020; Yoshida et al. 2020). Phylogenetic analysis showed that the gene number of GA2oxs expanded and evolved independently after the divergence of eudicots and monocots (Han and Zhu 2011; Kawai et al. 2014; Takehara et al. 2020; Yoshida et al. 2020). The rapid expansion of GA2oxs in eudicots and monocots was thought to be due to large-scale genome duplications (Huang et al. 2015); the duplicated genes may have either lost their function or retained their function, but their promoter and/or coding sequences diverged over time to form genes with various expression patterns and functions in the family (Panchy et al. 2016).

The duplicated genes that lost their function could have been the result of nonfunctional mutations in coding regions that resulted in premature stop codons and/or shifts in reading frame or in intro-exon structure (Balakirev and Ayala 2003), or the loss of function also could have been the result of sequence mutations within the promoter region that deactivated the expression, which could also lead to loss of its function; both of these are processes of gene pseudogenization (Yang et al. 2011). In addition, genome-wide comprehensive analysis of pseudogenes in rice and Arabidopsis showed that the coding regions of these pseudogenes tend to present increased amounts of nonsynonymous substitutions (Zou et al. 2009). Other than encountering pseudogenization, the duplicated genes in the family would also encounter sequence divergence in the regulatory and/or coding regions that caused RNA expression and protein function divergence, causing these genes to undergo either regulatory hypofunctionalization or subfunctionalization (Duarte et al. 2006; Panchy et al. 2016). Regulatory hypofunctionalization is where one member of a paralogous pair has an overall decrease in expression level while maintaining its protein function (Duarte et al. 2006). The expression difference between duplicated genes is a common phenomenon in plants, as 70% of duplicated gene pairs from Arabidopsis have different expression profiles (Ganko et al. 2007), and this phenomenon has also been observed in rice (Li et al. 2009) and cotton (Renny-Byfield et al. 2014). This divergence in expression among duplicated genes is negatively correlated with nonsynonymous substitution rates in the coding region (Li et al. 2005; Ganko et al. 2007), meaning that divergence in expression tends to evolve at the time when protein function becomes conserved in a family. The presence of duplicated genes retaining functional redundancy but with reduced expression could represent a way to maintain duplicates in a family (Qian et al. 2010). Additionally, duplicated genes could function as genetic buffers to help plants overcome various environmental stresses (Duarte et al. 2006; Zhang 2012; Illouz-Eliaz et al. 2019).

Like all other duplicated genes in a family, each GA2ox gene is differentially expressed in various tissues at different growth stages (Rieu et al. 2008; Giacomelli et al. 2013; Chen et al. 2016; Li et al. 2019), indicating that the divergence in expression of each individual GA2ox gene corresponds to different evolved biological functions. For example, *SlGA2ox7*, which is highly expressed in the stems (Chen et al. 2016), was suggested to be responsible for the regulation of stem-specific elongation in tomato (Schrager-Lavelle et al. 2019); *MtGA2ox10*, which is expressed in symbiotic tissues and nodules, was proposed to be involved in the regulation of rhizobial infection and nodule development in *M. truncatula* (Kim et al. 2019); and in rice, *OsGA2ox1* expressed around the shoot apex was proposed to be involved in the regulation of phase transition (Sakamoto et al. 2001), whereas *OsGA2ox3* was homeostatically regulated by bioactive GAs, which was suggested to play an important role in GA homeostasis (Sakai et al. 2003). Moreover, the expression of *OsGA2ox4* is upregulated by light and affects internode elongation, which might contribute to lodging resistance (Hirose et al. 2012; Liu et al. 2018). These expressional and functional correlations could have been evolved from sequence divergences between duplicated copies in those families (Li et al. 2005; Moghe and Last 2015).

Indeed, differential RNA expression resulting from sequence differences in the regulatory regions in many hormone-responsive genes and GA biosynthetic and catabolic genes has been observed. For example, within the Arabidopsis *GA2ox* gene family, the *AtGA2ox6* promoter contains the AGL15 binding motif (CCAATTTAATGG) and the absence of AGL15 reduces *AtGA2ox6* expression (Wang et al. 2004), whereas the *AtGA2ox7* promoter contains a DRE-like motif (GCCGAC and ATCGAC), and the expression of this gene is strongly upregulated during high-salinity stress (Magome et al. 2008). In addition, the expression of *AtGA2ox2 and AtGA2ox4* is upregulated by KN1-like homeobox (KNOX) proteins to reduce bioactive GA levels in the shoot apical meristem (SAM) (Jasinski et al. 2005). Similarly, in maize, *ZmGA2ox1* contains a KNOTTED1 (KN1) binding site in the first intron that is regulated by KNOX and is responsible for the bioactive GA levels around the SAM (Bolduc and Hake 2009). In sorghum, several cis-regulatory elements related to ABA and GA signaling were identified in the *SbGA2ox3* promoter region responsible for different degrees of dormancy between dormant IS9530 and less dormant Redland B2 grains (Rodriguez et al. 2012; Cantoro et al. 2013). The SD1^C9285^ (*OsGA20ox2* in C9285) haplotype includes 17 specific polymorphisms in the promoter and second intron region that affect the differential expression of *OsGA20ox2* between C9285 and Taichung 65 under submergence or ethylene treatment (Kuroha et al. 2018). Moreover, a comprehensive report showed that sequence-specific response elements in the regulatory regions are highly conserved in many hormone-responsive genes among angiosperms to regulate their hormone responses, and any replacement of the conserved sequences affected their tissue-specific expression (Lieberman-Lazarovich et al. 2019).

The functional divergence among genes in a family might also have evolved from amino acid sequence variations (Moghe and Last 2015; Panchy et al. 2016). Several studies have shown that amino acid sequence differences in rice GA2ox and GA20ox significantly influence their protein activities. Our previous study demonstrated that different amino acid replacements in OsGA2ox6 affected GA deactivation activities in plants to varying degrees (Lo et al. 2017), and a single sequence variation in the Shortened Basal Internodes (SBI, OsGA2ox4) allele that caused an amino acid substitution at position G338R of OsGA2ox4 significantly affected its activity (Liu et al. 2018). The crystal structure of OsGA2ox3 shows that the amino acid C194 is crucial for tetramer formation and is responsible for the approximately 9-fold *Km* and 4-fold *V*max values of the monomer, and this C194 amino acid was conserved only in OsGA2ox3, 4 and 8 but diverged in other members in the family (Takehara et al. 2020). Another study showed that an OsGA20ox2 allele in the deep-water rice variety C9285 has two amino acid substitutions that lead to a 271-fold increase in activity compared with that in Taichung 65, and this substitution, which is functionally distinct, naturally evolved and was then selected by breeders for deep-water rice cultivation (Kuroha et al. 2018).

In rice, although the biological roles of *OsGA2ox1*, *3*, and 4 corresponding to their specific expression patterns have been characterized (Sakamoto et al. 2001; Sakai et al. 2003; Liu et al. 2018), no comprehensive comparison of expression profiles for all *OsGA2oxs* has been undertaken. Moreover, since the substrate preference of GA2ox proteins could be different among the members of the family (Lange and Lange 2020) and their GA deactivation capability could also vary by amino acid sequence divergence (Lo et al. 2017; Liu et al. 2018; Takehara et al. 2020), to evaluate the functional correlation and importance for each *OsGA2ox* gene in the family, apart from the expression profiles, additional determinants such as their sequence differences in the regulatory and coding regions as well as their GA deactivation capability should be included.

Gene functional studies rely mostly on loss-of-function knockout mutants; however, knockout mutants of one member of the family usually show no discernible phenotype due to the functional redundancy existing among other members of that family (Rutter et al. 2017). Therefore, for a family of 10 genes, such as the rice GA2ox family, exploring the function of each individual gene through a knockout mutant approach is not always feasible. Thus far, only a limited number of single *GA2ox* knockout mutants in tomato C_20_-type *SlGA2ox7* (Schrager-Lavelle et al. 2019), *M. truncatula* C_20_-type *MtGA2ox10* (Kim et al. 2019) and rice *OsGA2ox3* (Takehara et al. 2020) in a large family with discernible phenotypes have been reported. In contrast, T-DNA activation-tagged mutants that lead to activation of a target gene that causes obvious phenotypic changes can facilitate the functional study of *OsGA2ox* genes (Lo et al. 2008). In addition to T-DNA activation-tagged mutants, transgenic plants ectopically overexpressing *OsGA2ox* genes could cause a quantifiable reduced-plant height phenotype and have been used to study their functional effects *in planta* (Lo et al. 2017). This overexpression approach that causes a quantifiable dwarf phenotype not only can be used to identify the functional effects of each gene but also can be used to compare all their GA deactivation capabilities *in planta* by quantifying the degree to which they reduce plant height.

In the present study, other than four previously characterized (*OsGA2ox3*, *5*, *6* and *9* genes) mutants (Lo et al. 2008), five additional (*OsGA2ox1*, *2*, *4*, *7* and *8* genes) uncharacterized T-DNA activation-tagged mutants were analyzed and used to facilitate the cloning of the full-length cDNA of their respective genes. In addition, transgenic plants overexpressing the full-length cDNA of each of the *OsGA2ox* genes ectopically in the same rice variety were compared to investigate their effects on plant height reduction, and their reducing effects were scored on the basis of relative plant height (RPH) to estimate their GA deactivation capability *in planta*. The importance and significance of four amino acids conserved in clade A but not in clade B of class I GA2oxs, which are crucial for GA deactivation, have been demonstrated by transgenic approaches *in planta*. In addition, through large-scale sequence comparisons of rice GA2ox genes from 4276 rice accessions (Zhao et al. 2015) and the GA2ox expression profiles collected from available databases (UniVIO: http://univio.psc.riken.jp/; RED IC4R: http://ic4r.org; RiceXPro; https://ricexpro.dna.affrc.go.jp/), as well as analysis of the GA deactivation capability of transgenic plants, three dominant paralogs in the family in each of their respective classes were revealed.

## Results

### Functional Screening of Rice *GA2ox1*, *GA2ox2*, *GA2ox4*, *GA2ox7* and *GA2ox8* Using T-DNA Activation-Tagged Mutants

Our previous study showed that enhanced expression of *OsGA2ox3* (M77777), *OsGA2ox5* (M27337), *OsGA2ox6* (M47191) and *OsGA2ox9* (M58817) in their respective T-DNA activation-tagged mutants reduced plant height, which provided a useful tool for functional screening of GA2ox genes (Lo et al. 2008). To complete the functional screening of all GA2ox genes in the family, five additional T-DNA activation-tagged mutants, M36548 (for *OsGA2ox1*), M43852 (for *OsGA2ox2*), M96803 (for *OsGA2ox4*), M66925 (for *OsGA2ox7*) and M61685 (for *OsGA2ox8*), were selected from the Taiwan Rice Insertional Mutant (TRIM) library (Hsing et al. 2007) through a reverse genetic approach.

**Fig. 1:**
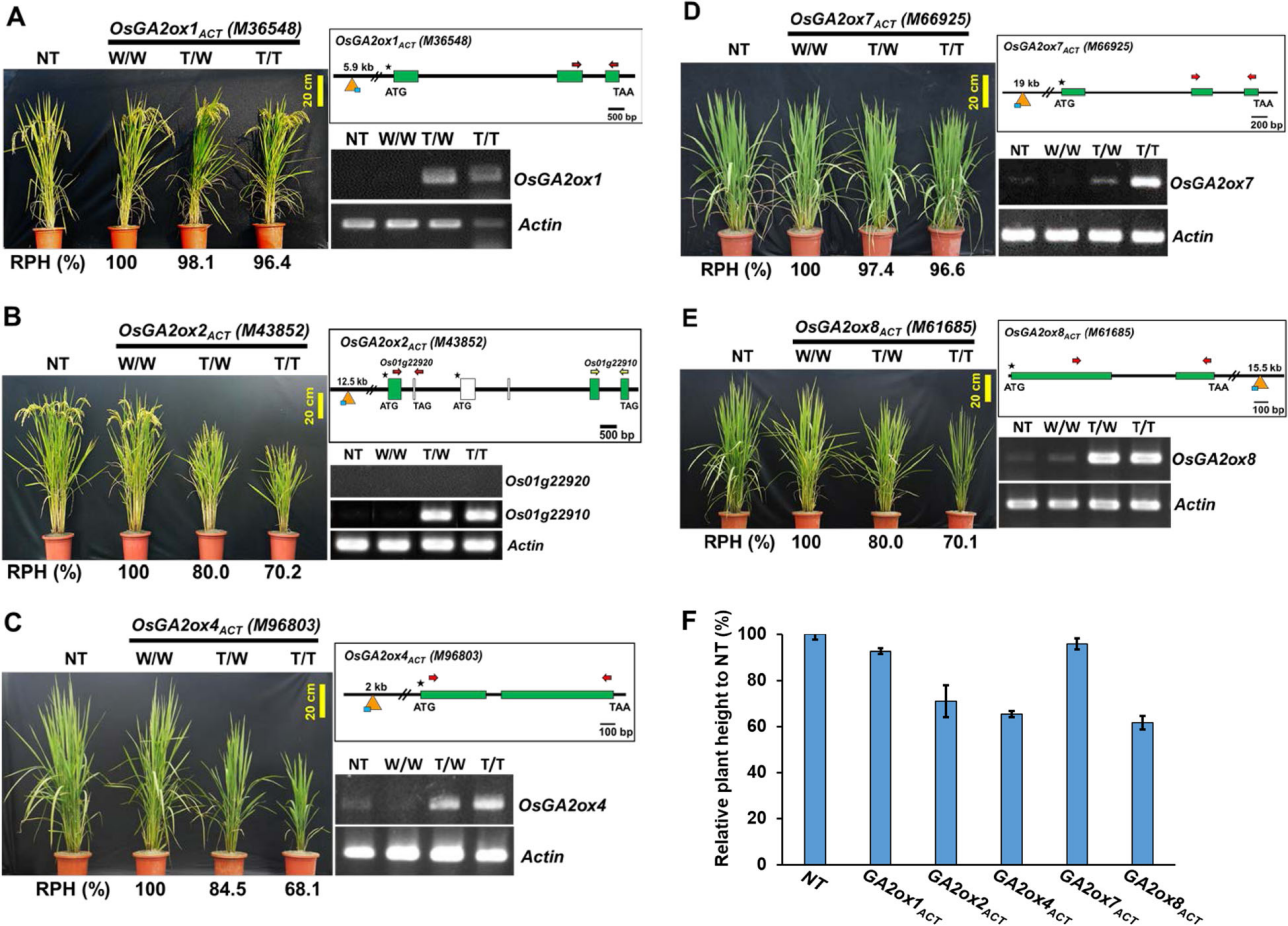
Phenotypes and expression characterization of the T-DNA activation-tagged mutants for *OsGA2ox1*, *2*, *4*, *7*, and *8.* The phenotypes and relative plant height (RPH %) of T-DNA activation-tagged mutants (with various genotypes (W/W, segregated WT; T/W, heterozygous; T/T, homozygous)) compared with those of nontransgenic plants (NT) and the RT-PCR analysis results of target gene activation are shown. The Actin gene was used as a control. The T-DNA insertion sites (orange triangles) relative to the ATG start codon of their target genes are shown in the graphic adjacent to the images. **a** The T-DNA mutant M36548 (an *OsGA2ox1*_*ACT*_ mutant), with the T-DNA inserted 5.9 kb upstream from *OsGA2ox1*, is shown. **b** The T-DNA mutant M43852 (an *OsGA2ox2*_*ACT*_ mutant), with the T-DNA inserted 12.5 kb upstream from *OsGA2ox2*, is shown. **c** The T-DNA mutant M96803 (an *OsGA2ox4*_*ACT*_ mutant), with the T-DNA inserted 2 kb upstream from *OsGA2ox4*, is shown. **d** The T-DNA mutant M66925 (an *OsGA2ox7*_*ACT*_ mutant), with the T-DNA inserted 19 kb upstream from *OsGA2ox7*, is shown. **e** The T-DNA mutant M61685 (an *OsGA2ox8*_*ACT*_ mutant), with the T-DNA inserted 15.5 kb downstream from *OsGA2ox8*, is shown. **f** RPH comparison of the homozygous plants of the above T-DNA activation-tagged mutants. Values are means ± SE (*n* = 10)

Among them, the T-DNA in mutant M36548 was inserted 5.9 kb upstream from *OsGA2ox1* (Fig. 1a), and the T-DNA in mutant M43852 was inserted approximately 12.5 kb upstream from a putative *OsGA2ox2* (LOC_Os01g22920) (Fig. 1b). In mutants M96803 (*OsGA2ox4*), M66925 (*OsGA2ox7*) and M61685 (*OsGA2ox8*), their T-DNAs were inserted either 2 kb upstream, 19 kb upstream or 15.5 kb downstream from their respective target *OsGA2ox* genes (Fig. 1c-e). Varying degrees of plant height reduction ranging from approximately 30% (70% RPH for mutants M43852, M96803 and M61685) to 4% (96% RPH for mutants M36548 and M66925) in their respective homozygous mutant lines were observed (Fig. 1f), suggesting that the genes were all functional. The difference in plant height-reducing effects among the mutants might be caused by various GA deactivation capabilities and/or different degrees of target gene activation among them.

To support the phenomenon that plant height-reducing effects in mutants are correlated with the activation of their target genes, the RNA expression of each *OsGA2ox* target gene in the T1 progeny, including segregated wild-type (W/W), heterozygous (T/W) and homozygous (T/T) plants of their respective mutants, was analyzed (Fig. 1a-e, top row of each gel). Except for the putative *OsGA2ox2* gene (LOC_Os01g22920), the results showed that the expression of each target gene in their respective mutants was activated and correlated with their reduced-plant height phenotypes. Although expression of the LOC_Os01g22920 gene was not detected, reduced height of its respective mutant M43852 was observed (Fig. 1b), suggesting that the annotation of LOC_Os01g22920 for *OsGA2ox2* was not correct or that the reduced-plant height phenotype might be caused by other unidentified T-DNA insertion events.

### The *OsGA2ox2* Gene Contains a Long First Intron and Three Exons that Encode 370 Amino Acids, with Two Typical Conserved Protein Domains

**Fig. 2:**
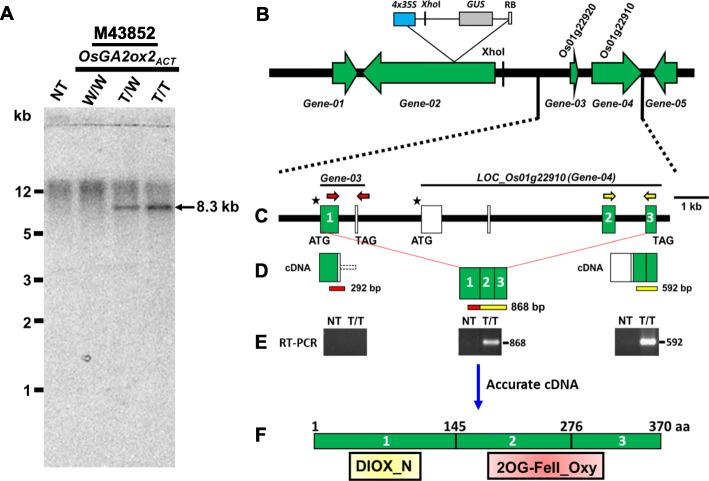
Southern blot assay of mutant M43852 and a schematic illustration of the RT-PCR results and possible cDNA and exon/intron structures to reannotate the gene structure of *OsGA2ox2*. **a** Southern blot assay showing only one copy of the T-DNA insertion in mutant M43852. **b** A schematic diagram showing five genes (named gene-01 to gene-05) annotated in a 40 kb region flanking the T-DNA insertion site. **c** Enlarged regions of annotated gene-03 (LOC_Os01g22920) and gene-04 (LOC_Os01g22910) with their possible exon (box) and intron (bold line) structures. The two primer sets (arrows) utilized to detect gene expression are shown above their genes. **d** cDNAs and expected sizes of RT-PCR products using the corresponding primer sets for the annotated gene-03, gene-04 and reannotated gene *OsGA2ox2*. **e** RT-PCR results of gene-03 (using the red primer set), gene-04 (using the yellow primer set that amplify 592 bp) and the reannotated gene *OsGA2ox2* (using the 5′ red and 3′ yellow primer sets that amplify 868 bp). **f** Accurate cDNAs for the reannotated OsGA2ox2. Three exons with 370 amino acids and the relative locations of the DIOX_N and 2OG-FeII_Oxy conserved protein domains

To confirm that the mutant M43852 contained no extra T-DNA insertions, a Southern blotting assay using *Xho*I-digested genomic DNA hybridized by a ^32^P-labeled GUS DNA probe was performed (Fig. 2a). The results showed that mutant M43852 contained only one copy of T-DNA inserted 12.5 kb upstream from LOC_Os01g22920, which was revealed by the expected 8.3 kb hybridized signal (Fig. 2a). The gene structure of the putative *OsGA2ox2*-LOC_Os01g22920 and the flanking genes 20 kb up- and downstream from the T-DNA insertion site were then checked, and five genes, named gene-01 to gene-05, including the putative *OsGA2ox2* (LOC_Os01g22920 is gene-03), were annotated in this 40 kb region (Fig. 2b). A series of schematic diagrams are provided to show the relative locations of these annotated genes, their transcription directions and the location of the T-DNA insertions (Fig. 2b). Also shown is an enlarged region containing the exons/introns, the start codon (ATG) and stop codon (TAG) of annotated gene-03 and gene-04 (LOC_Os01g22910) (Fig. 2c), and their proposed spliced cDNA with the expected size of RT-PCR products (Fig. 2d) using the primer sets indicated with colored arrows (Fig. 2c).

Among these flanking genes, LOC_Os01g22910 was located downstream of LOC_Os01g22920 and was strongly activated in mutant M43852 (Figs. 1b; 2e), and this gene activation was associated with a reduction in plant height (Fig. 1b). Although LOC_Os01g22910 was annotated as a retrotransposon protein (Kawahara et al. 2013), its deduced protein contained a 2OG-FeII_Oxy domain, one of the conserved domains of GA2ox (Fig. 2f). GA 2-oxidase belongs to the DOXC class of the 2OGD superfamily, whose members contain a conserved DIOX_N domain (Pfam ID: PF14226) at the N-terminal region and a 2OG-FeII_Oxy domain (Pfam ID: PF03171) at the carboxyl terminus (Kawai et al. 2014). The deduced protein of LOC_Os01g22920 (gene-03, putative OsGA2ox2) contains a DIOX_N domain but no 2OG-FeII_Oxy domain in its C-terminal region (Han and Zhu 2011; Liu et al. 2018), and its downstream gene LOC_Os01g22910 contains a 2OG-FeII_Oxy domain, implying that LOC_Os01g22910 is part of OsGA2ox2.

**Fig. 3:**
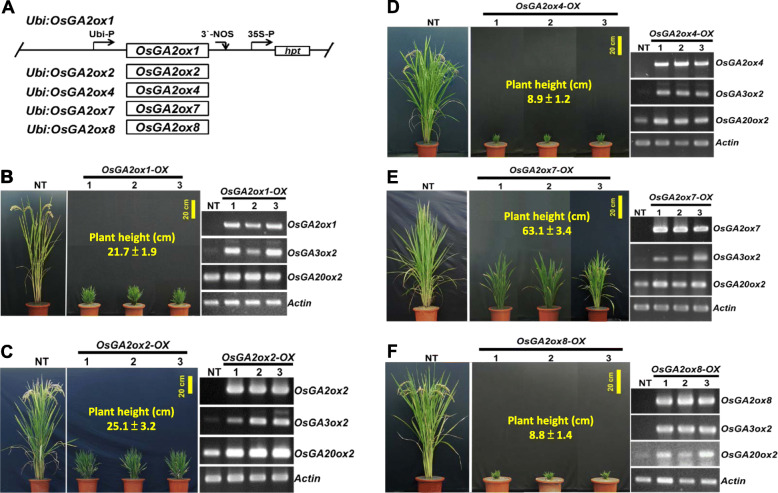
Phenotypic comparisons and characterization of *OsGA2ox1*, *2*, *4*, *7*, and *8* expressions in overexpression transgenic rice plants*.* **a** Schematic diagram showing the overexpression constructs of *Ubi:OsGA2ox1*, *Ubi:OsGA2ox2*, *Ubi:OsGA2ox4*, *Ubi:OsGA2ox7*, and *Ubi:OsGA2ox8.* The full-length cDNA from each gene was cloned into the same vector construct. Three representative T_0_ transgenic plants from each of the *OsGA2ox1-OX* (**b**), *OsGA2ox2-OX* (**c**), *OsGA2ox4-OX* (**d**), *OsGA2ox7-OX* (**e**), and *OsGA2ox8-OX* (**f**) transgenic lines were compared to their nontransgenic (NT) plants. The average plant height in cm (means ± SE, n≧10) for each gene constructs are shown. RT-PCR was performed to analyze the respective target genes and marker genes of *OsGA3ox2* and *OsGA20ox2*, and the Actin gene (as an internal control) is shown

To verify the above implications, RT-PCR analysis for mutant M43852 with various primer combinations was performed. While an expected 292 bp signal (Fig. 2d) with a primer set based on the gene-03 annotation (Fig. 2c) was not detected (Fig. 2e, the same RT-PCR result shown in Fig. 1b), the 592 bp signal with a primer set based on the gene-04 annotation and the 868 bp signal (Fig. 2d) with a primer set for exons #1 and #3 of the new annotation, including a combination of gene-03 and gene-04 with three misannotated exons (empty boxes) as part of the first long ~ 8 kb intron (Fig. 2c), was strongly activated (Fig. 2e). With these RT-PCR results, we reannotated this *OsGA2ox2* gene and proposed that this Os*GA2ox2* gene is the combination of LOC_Os01g22920 and LOC_Os01g22910, with three exons (green boxes) and a long first intron (Fig. 2c). This annotation was further confirmed by an *OsGA2ox2* cDNA clone isolated from the OsGA2ox2-activated T-DNA mutant M43852 and functionally identified by overexpressing *OsGA2ox2* in transgenic rice plants (Fig. 3c). We therefore concluded that this Os*GA2ox2* gene contains a long first intron and three exons and encodes 370 amino acids, with two typical conserved DIOX_N and 2OG-FeII_Oxy protein domains (Fig. 2c, f), and the gene is functional.

### Functional Identification of Rice *GA2ox1*, *GA2ox2*, *GA2ox4*, *GA2ox7* and *GA2ox8* with Overexpression Transgenic Plants

In the rice GA2ox gene family, *OsGA2ox3* and *OsGA2ox 5*, *6*, and *9* (C_20_-type GA2ox genes) were characterized in our previous study by overexpressing them in transgenic plants (Lo et al. 2008). To complete the functional identification of all the members of the rice GA2ox gene family, the full-length cDNAs of the other *OsGA2ox* genes, including *GA2ox1*, *GA2ox2*, *GA2ox4*, *GA2ox7* and *GA2ox8*, were cloned from their respective T-DNA activation-tagged (*OsGA2oxs*_ACT_) mutants (Fig. 1) and expressed ectopically in TNG67 driven by the maize ubiquitin (Ubi) promoter construct *Ubi:OsGA2oxs* (Fig. 3a), the same vector used in all our previous studies (Lo et al. 2008; Lo et al. 2017).

Three independent transgenic rice lines with each *Ubi:OsGA2ox* transformation were generated, and the RNA expression of their target *OsGA2ox* genes was visualized (Fig. 3b-f, and the top row of each gel). As expected, dwarf phenotypes were observed for all *OsGA2ox* overexpression (*OsGA2oxs-OX*) transgenic plants. *OsGA2ox1-OX* and *OsGA2ox2-OX* showed moderate reductions (Fig. 3b, c), *OsGA2ox4-OX* and *OsGA2ox8-OX* showed severe reductions (Fig. 3d, f), and *OsGA2ox7-OX* showed minor reductions (Fig. 3e) in height. These varying degrees of plant height reduction suggested that the GA deactivation capability varied. To explore whether the plant height reduction in *OsGA2oxs-OX* transgenic plants resulted from GA deficiency, the expression of two GA deficiency-regulated biosynthetic genes, *OsGA3ox2* and *OsGA20ox2*, was investigated, and the expression of both of them was enhanced in all examined *OsGA2oxs-OX* transgenic rice lines (Fig. 3b-f, the middle two rows of the gel). In addition, the dwarf seedlings germinated from the two seed-bearing lines (*OsGA2ox2-OX* and *OsGA2ox7-OX*), and their phenotype could be rescued by exogenous GA_3_ treatment (Additional file: Fig. S2).

### *OsGA2ox7-OX* Transgenic Rice Plants Revealed a Distinct Semidwarf Phenotype Different from that of the Other *OsGA2oxs*-OX Transgenic Plants

**Fig. 4:**
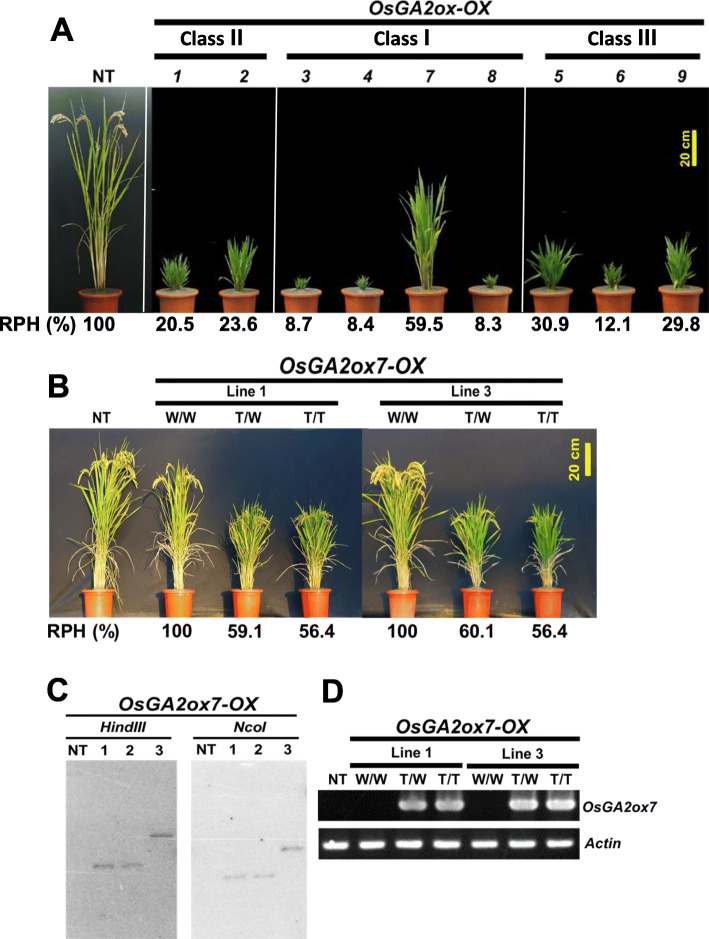
Phenotypic comparison of the transgenic rice plants overexpressing all the rice GA2ox genes and characterization of the *OsGA2ox7-OX* transgenic lines. **a** Phenotypic comparison of the transgenic rice plants overexpressing for all rice GA2ox genes. A representative T_0_ transgenic plant overexpressing each of the 9 rice GA2ox genes under the same comparison basis is shown. The plants were grown in a greenhouse. **b** Phenotypic comparison of the *OsGA2ox7-OX* overexpression transgenic lines. The insertion events of lines 1 and 3 were identified, and their phenotypes vs genotypes (W/W no T-DNA; T/W hetero; T/T homo) and RPH compared with that of the NT plants are shown. The plants were grown in paddy fields. **c** Southern blot assay showing a single T-DNA insertion for all three lines. The line-1 and line-3 showing independent insertion events. **d** Results of RT-PCR analysis of various genotypes of *OsGA2ox7-OX* transgenic line-1 and line-3. The Actin gene was used as a control

To verify whether each member of the *OsGA2ox* family showed various GA deactivation capabilities, transgenic plants overexpressing each *OsGA2ox* gene ectopically in the TNG67 rice variety with the same vector construct were obtained and pooled together for comparison (Fig. 4a). The *OsGA2ox* overexpression transgenic plants in class II and class III presented approximately 12.1% to 30.9% RPH compared with that of the nontransgenic (NT) plants. Unlike the severe dwarf with approximately 8.3% to 8.7% RPH caused by the *OsGA2ox3*, *4* and *8* genes, the *OsGA2ox7-OX* in the same class (I) resulted in approximately 59.5% RPH (Fig. 4a), which garnered our attention for further investigation.

These distinct semidwarf *OsGA2ox7-OX* transgenic plants were further evaluated with two independent lines (Fig. 4b), each with one copy of a T-DNA insertion confirmed by Southern blotting assays (Fig. 4c). The T-DNA that inserted into the long arm of chromosome #12 for line-1 and into the short arm of chromosome #3 for line-3 were identified, and the RPH (%) of their corresponding genotypes (Fig. 4b), along with high levels of *OsGA2ox7* expression, were stably inherited in their progeny (Fig. 4d). The agronomic trait data of the transgenic plants, compared with the NT plants, presented approximately 59–61% RPH for heterozygous (T/W) plants, 55–56% RPH for homozygous (T/T) plants, a 30% increase in tiller number and a panicle length of approximately 80% during the spring 2020 season (Table 1).

**Table 1:** Agronomic traits comparisons between OsGA2ox7-OX transgenic lines and NT/TNG67 plants^#^

Rice plants		Plant height (cm)	Heading date (DAI)	Tiller number	Panicle number	Panicle length (cm)	1000-grain weight (g)
**NT/TNG67**		**114.0 ± 4.6**^a^	**115.2 ± 1.2**^a^	**14.6 ± 1.1**^a^	**14.0 ± 2.0**^a^	**16.5 ± 1.2**^a^	**25.4 ± 0.6**^a^
**Line-1**	**T/W**	**65.2 ± 1.8**^b^	**117.8 ± 1.3**^b^	**19.4 ± 1.8**^b^	**18.2 ± 2.9**^b^	**13.5 ± 0.7**^b^	**23.3 ± 0.8**^b^
	**T/T**	**61.6 ± 2.1**^c^	**118.1 ± 1.1**^b^	**19.6 ± 2.7**^b^	**18.6 ± 2.4**^b^	**13.4 ± 0.5**^b^	**22.8 ± 1.0**^b^
**Line-3**	**T/W**	**67.8 ± 1.3**^b^	**117.4 ± 1.2**^b^	**19.4 ± 2.9**^b^	**19.0 ± 2.7**^b^	**13.5 ± 0.5**^b^	**22.6 ± 1.1**^b^
	**T/T**	**62.4 ± 3.3**^c^	**118.3 ± 1.3**^b^	**20.2 ± 2.8**^b^	**19.6 ± 2.7**^b^	**14.3 ± 0.7**^b^	**23.5 ± 0.6**^b^

The different letters indicate significant differences between samples according to the Turkey’s HSD test (*p* < 0.05). NT/TNG67: rice variety TNG67; T/W heterozygous, T/T homozygous for transgene

*DAI* days after imbibition

^#^The values are the means ± SEs (*n* = 5)

### Phylogenetic Analysis of Class I *GA2ox* Genes in the Poaceae Revealed Two Functionally Distinct Clades

**Fig. 5:**
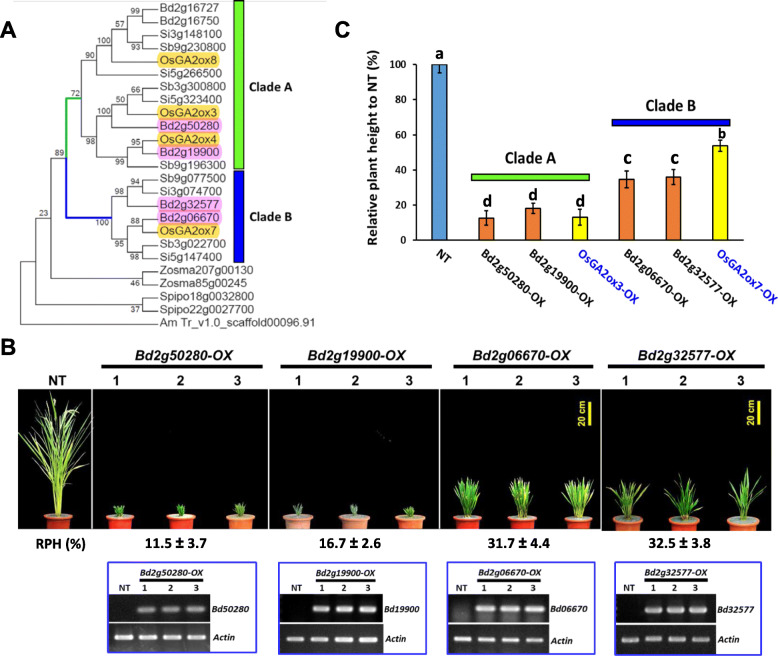
Two functionally distinct clades of class I *GA2ox* genes in monocots according to phylogenetic analysis and their functional identifications. **a** Phylogenetic analysis of class I GA2ox genes in monocots revealed two functionally distinct clades. **b** Functional identification of orthologous genes of *Bd2g50280* (orthologous to *OsGA2ox3*), *Bd2g19900* (orthologous to *OsGA2ox4*), *Bd2g06670* (orthologous to *OsGA2ox7*), and *Bd2g32577* (orthologous to *OsGA2ox10*) from *B. distachyon.* Three representative T_0_ transgenic rice plants from each of the transgenic overexpression lines and their average RPH are shown. Values are means ± SE (*n* = 5). The RT-PCR analysis results for each target gene of three independent transgenic lines are shown. The Actin gene was used as a control. **c** Statistical comparisons of RPH between clades A and B as a result of the orthologous genes from *B. distachyon* and rice. Values are means ± SE (*n* = 5). The different letters indicate significant differences between samples according to the Turkey’s HSD test (*p* < 0.05)

The plant height-reducing effect of *OsGA2ox7* was much weaker than that of other members in class I, which led us to compare whether any variation existed among class I genes that might cause this difference. The sequences of all available *GA2ox* genes that were categorized into class I were collected and analyzed. A phylogenetic tree comprising class I *GA2ox* genes from *Brachypodium distachyon* (*Bd*), *Oryza sativa* (*Os*), *Setaria italica* (*Si*), *Sorghum bicolor* (*Sb*), *Spirodela polyrhiza* (*Sp*) and *Zostera marina* (*Zm*) was generated. In the phylogenetic tree, the class I GA2oxs of Poaceae species could be further divided into clade A and clade B (Fig. 5a). The *ZmGA2oxs* and *SpGA2oxs* were not included in either clade A or clade B, suggesting that both clades formed after the divergence of the early branching of monocots but before Poaceae species diverged. Clade B included orthologs of *OsGA2ox7* and *OsGA2ox10*; however, *OsGA2ox10* was excluded from the tree due to its absence of the DIOX_N domain (Fig. 5a).

Unlike in clade B genes, overexpression of clade A genes (*OsGA2ox3*, *4* and *8*) caused a significant effect on growth suppression of transgenic rice plants (Fig. 4a). To further confirm whether the growth suppression effect was different between clade A and clade B, orthologs of *OsGA2ox3* (*Bd2g50280*), *OsGA2ox4* (*Bd2g19900*), *OsGA2ox7* (*Bd2g06670*), and *OsGA2ox10* (*Bd2g32577*) from *B.distachyon* were ectopically overexpressed in transgenic rice plants. Three independent lines revealed the same RNA expression levels from each *BdGA2oxs-OX* transgenic rice plant were compared, and *Bd2g50280-OX* and *Bd2g19900-OX* showed severe dwarf phenotypes (Fig. 5b) similar to those resulting from the rice clade A genes (Fig. 4a), while *Bd2g06670*-OX and *Bd2g32577*-OX transgenic rice plants revealed less growth suppression (Fig. 5b), the trait of which was similar to that caused by *OsGA2ox7*, a clade B gene (Fig. 4a). Unlike the *Bd2g50280-OX* and *Bd2g19900-OX* transgenic rice plants, the *Bd2g06670-OX* and *Bd2g32577*-OX transgenic rice plants produced viable seeds. This distinct functional effect on growth suppression of plants between clades A and B (Fig. 5c) within class I suggests that the GA deactivation capability may have been attenuated in clade B, which might have been caused by certain amino acid variations or structural differences in the members between these two clades.

### Four Amino Acid Variants in OsGA2ox3 Attenuated its Capability for GA Deactivation

**Fig. 6:**
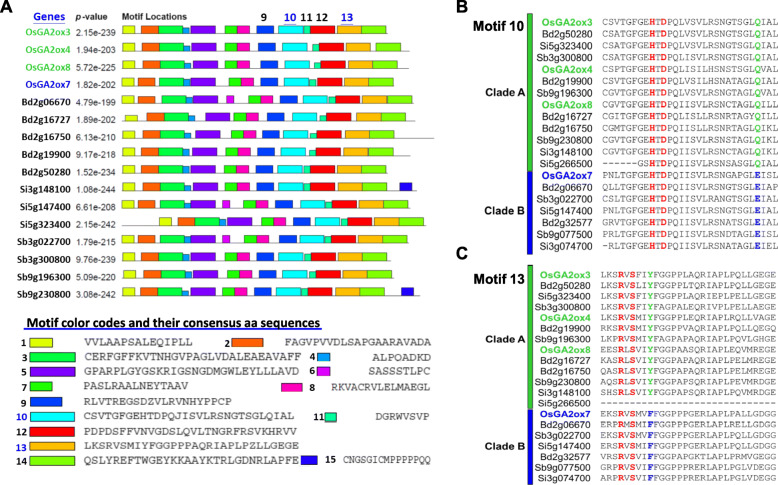
Conserved protein motif identification among class I GA2oxs from Poaceae species and identification of distinguishable amino acids between clades A and B. **a** Conserved protein motif identification among 16 class I GA2oxs from Poaceae species by MEME algorithm analysis. The alignment of the 15 conserved motifs indicated by color codes and the consensus amino acid sequences of each conserved motif are shown. **b** Detailed consensus amino acid sequence alignment of motif 10, distinguished by Q in clade A and E in clade B. A putative iron-binding site (HTD) is shown in red. **c** Detailed consensus amino acid sequence alignment of motif 13, distinguished by Y in clade A and F in clade B. A putative 2-OG-binding site is shown in red

To determine which amino acids might be responsible for the different GA deactivation capabilities between members of clades A and B of class I GA2oxs, the amino acid sequences of class I GA2oxs from Poaceae species were first analyzed by the MEME algorithm to identify conserved protein motifs (Bailey et al. 2015). A total of 15 conserved motifs were identified (Fig. 6a), and these motifs were further aligned to identify whether any significant amino acid variations occurred between clades A and B. Two distinguishable amino acid variants were revealed by sequence alignment comparing available sequences of clade A and B genes from *O. sativa* (*Os*), *B. distachyon* (*Bd*), *S. bicolor* (*Sb*) and *S. italica* (*Si*) (Fig. 6b, c). The first variant is Gln (Q220) in clade A vs Glu (E227) in clade B in motif 10 (Fig. 6b), and the second variant is Tyr (Y274) in clade A vs Phe (F283) in clade B in motif 13 (Fig. 6c); they were positioned near the iron (HTD)- and the 2-oxoglutarate (2OG)-binding sites (RVS) (Fig. 6b, c). A 3-D protein structure of OsGA2ox3 with accession number 6KU3 downloaded from the Protein Data Bank was used to show the relative positions of amino acids Q220 and Y274 in the catalytic pocket (Additional file: Fig. S3).

**Fig. 7:**
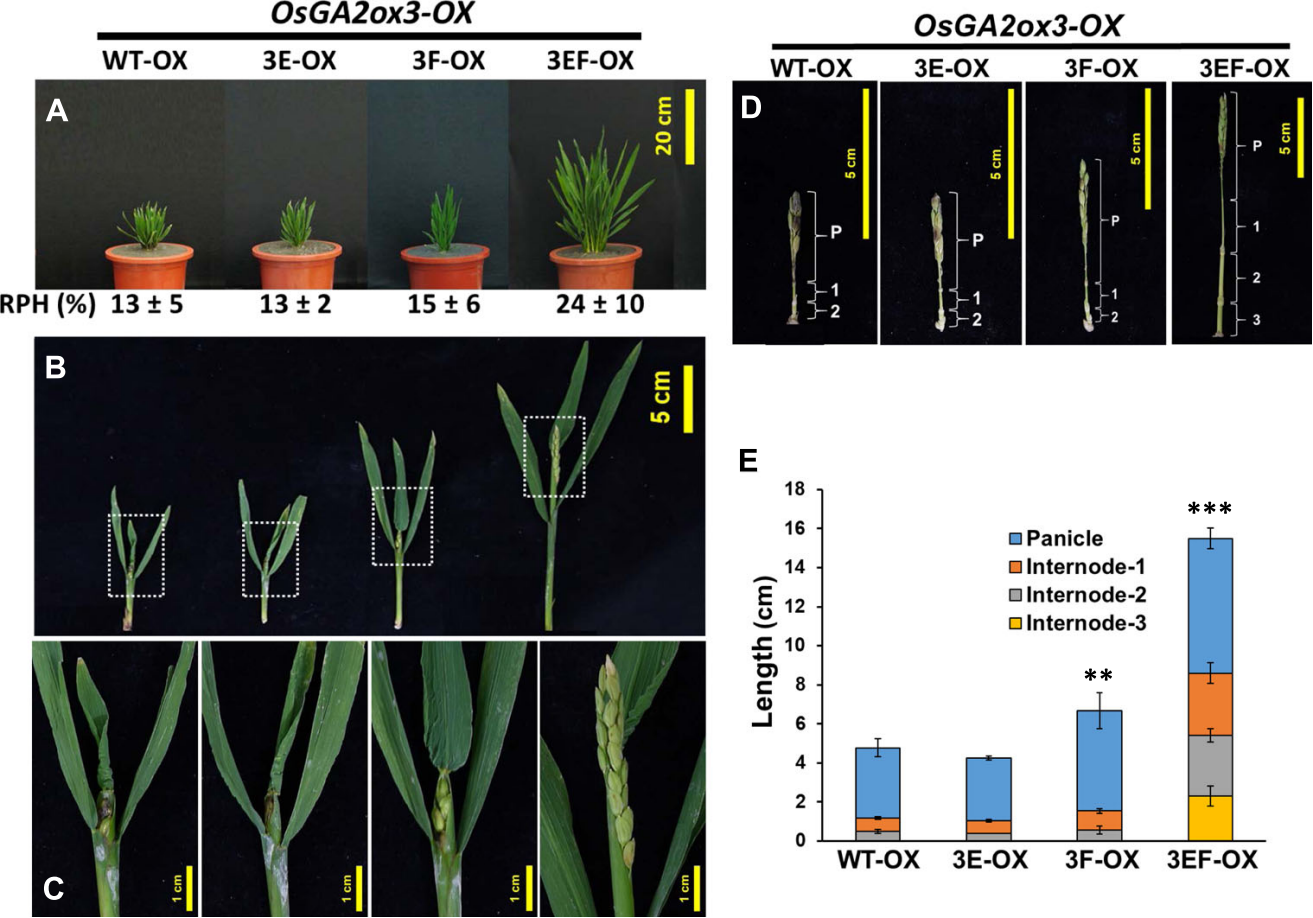
Phenotypic comparison of WT-OX, 3E-OX, 3F-OX and 3EF-OX transgenic rice plants*.* **a** Phenotypic comparison of WT-OX, 3E-OX, 3F-OX and 3EF-OX transgenic rice plants. A representative T_0_ transgenic plant with the average RPH resulting from each overexpression line is shown. Values are means ± SE (*n* = 5). **b** A dissected representative main culm from each overexpression plant is shown to compare their emerging panicles at the maturation stage. **c** Enlarged image from the boxed area of B) to show details. **d** Comparisons of the length of panicles and internodes from each overexpression line. P: panicle; 1: internode #1; 2: internode #2; 3: internode #3. **e** Statistical comparisons of the length of the main culms (including panicles and internodes) from each overexpression line. Values are means ± SE (*n* = 5). The significant differences using Student’s *t*-test are indicated by *p < 0.05, **p < 0.01, ***p < 0.001

To investigate whether these two amino acids were important and could attenuate the GA deactivation capability, the amino acids Q220 and Y274 in OsGA2ox3 were replaced with glutamic acid (E) and phenylalanine (F), respectively, or simultaneously to create OsGA2ox3E (3E), OsGA2ox3F (3F) and OsGA2ox3EF (3EF) mutants. These 3E, 3F, and 3EF mutant and wild-type (WT) OsGA2ox3 genes were expressed ectopically in transgenic rice with similar high RNA expressions (Additional file: Fig. S4) and were used for comparison. The overexpression transgenic plants *3E-OX* and *3F-OX* showed the same severe dwarf phenotype as their *WT-OX* transgenic plants did (Fig. 7a). Although some *3F-OX* transgenic plants were slightly taller, their height was not significantly different among the population. However, the double point mutated gene in the *3EF-OX* transgenic plants caused a significant plant height increase (Fig. 7a). Unlike the severely dwarf *3E-OX*, *3F-OX* and *WT-OX* transgenic plants, which showed no panicles, the *3EF-OX* transgenic plants showed protruding panicles (Fig. 7b, c) and produced some viable seeds. The culms were dissected to show that the relative length of the panicle and internodes (Fig. 7d) and the length of the panicle and all internodes in *3EF-OX* transgenic plants all increased (Fig. 7e), suggesting that the replacement of Q220E and Y274F in OsGA2ox3 attenuated its GA deactivation capability.

**Fig. 8:**
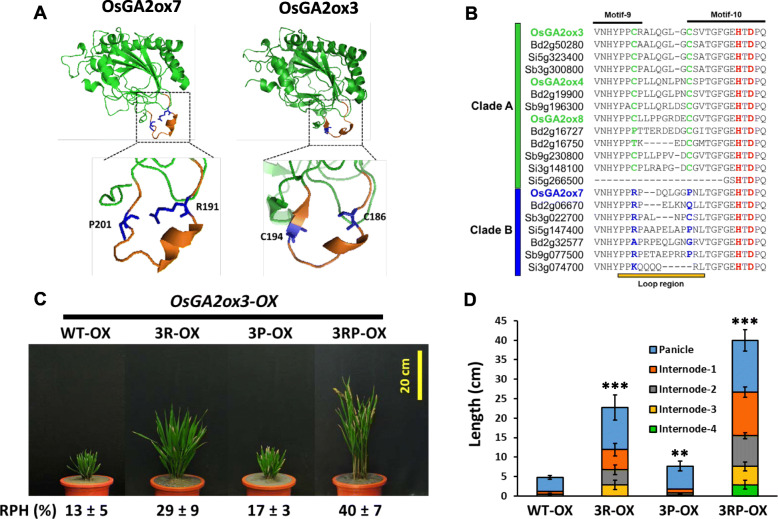
Comparisons of OsGA2ox7 and OsGA2ox3 3-D protein structures and sequence alignment to show the conserved amino acids between clades A and B and the phenotypes of WT-OX, 3R-OX, 3P-OX and 3RP-OX transgenic rice plants*.* **a** Simulated protein structure of OsGA2ox7 and published protein structure of OsGA2ox3 for comparison. The loop regions contain clade A-specific C186 and C194, and their corresponding amino acids R191 and P201 in OsGA2ox7 show different structures. The 3-D protein structures were visualized by using PyMOL (Schrodinger 2015). **b** Detailed consensus amino acid sequence alignment of the loop region between motif 9 and motif 10 distinguished by two conserved Cs (highlighted with green color) in clade A from other variants (highlighted with blue color) in clade B. **c** Phenotypic comparison of WT-OX, 3R-OX, 3P-OX and 3RP-OX transgenic rice plants. A representative overexpression T_0_ transgenic plant with the average RPH from each overexpression line is shown. Values are means ± SE (*n* = 5). **d** Statistical comparisons of the length of the main culms (including panicles and internodes) from each overexpression line. Values are means ± SE (*n* = 5). The significant differences using Student’s *t*-test are indicated by *p < 0.05, **p < 0.01, ***p < 0.001

Since the *3EF-OX* plants presented only approximately 24% RPH compared with that of the NT plants (Fig. 7a), which was not comparable to the approximately 60% RPH of the NT plants observed for *OsGA2ox7-OX* (Fig. 4a), this suggests that the attenuation of the GA deactivation capability of OsGA2ox7 could not be fully determined by the replacement of Q220 and Y274. To further investigate any other causal amino acid variants involved in attenuating GA deactivation capability, the protein structure of OsGA2ox7 was modeled by using the Phyre2 server (Kelley et al. 2015) and compared to the structure of OsGA2ox3 (Fig. 8a). A loop region that contains approximately 13–14 amino acids located between motif 9 and motif 10 showed a divergent structure between OsGA2ox3 and OsGA2ox7 (Fig. 8a, b). Sequence alignment between clades A and B revealed two distinguishable amino acid variants: a variant Cys (C186) in OsGA2ox3 vs Arg (R191) in OsGA2ox7 and another variant Cys (C194) in OsGA2ox3 vs Pro (P201) in OsGA2ox7 in this loop region (Fig. 8a).

To evaluate the impact of these amino acid variants on GA2ox activity, amino acids C186 and C194 of OsGA2ox3 were replaced with arginine (R) and proline (P), respectively, or simultaneously to create OsGA2ox3R (3R), OsGA2ox3P (3P) and OsGA2ox3RP (3RP) mutants. The transgenic plants overexpressed these mutated genes all at the same high levels (Additional file: Fig. S5). The transgenic plants overexpressing the 3P mutant (*3P-OX*) showed a slight plant height increase from 13% to approximately 17% RPH of that of the NT plants. However, *3R-OX* transgenic plants showed a greater RPH increase of up to approximately 29% of that of the NT plants, and the double point-mutated gene in the *3RP-OX* transgenic plants resulted in a significant RPH increase of up to approximately 40% to 47% of that of the NT plants (Fig. 8c). The *3R-OX and 3RP-OX* transgenic plants showed protruding panicles (Fig. 8c) and could produce viable seeds, with increased panicle and internode lengths (Fig. 8d). These observations suggested that the replacement of C186R and C194P in the loop region of OsGA2ox3 attenuated its GA deactivation capability.

### Analysis of Gene Structure and Sequence Differences Suggest that *OsGA2ox10* is a Pseudogene

**Fig. 9:**
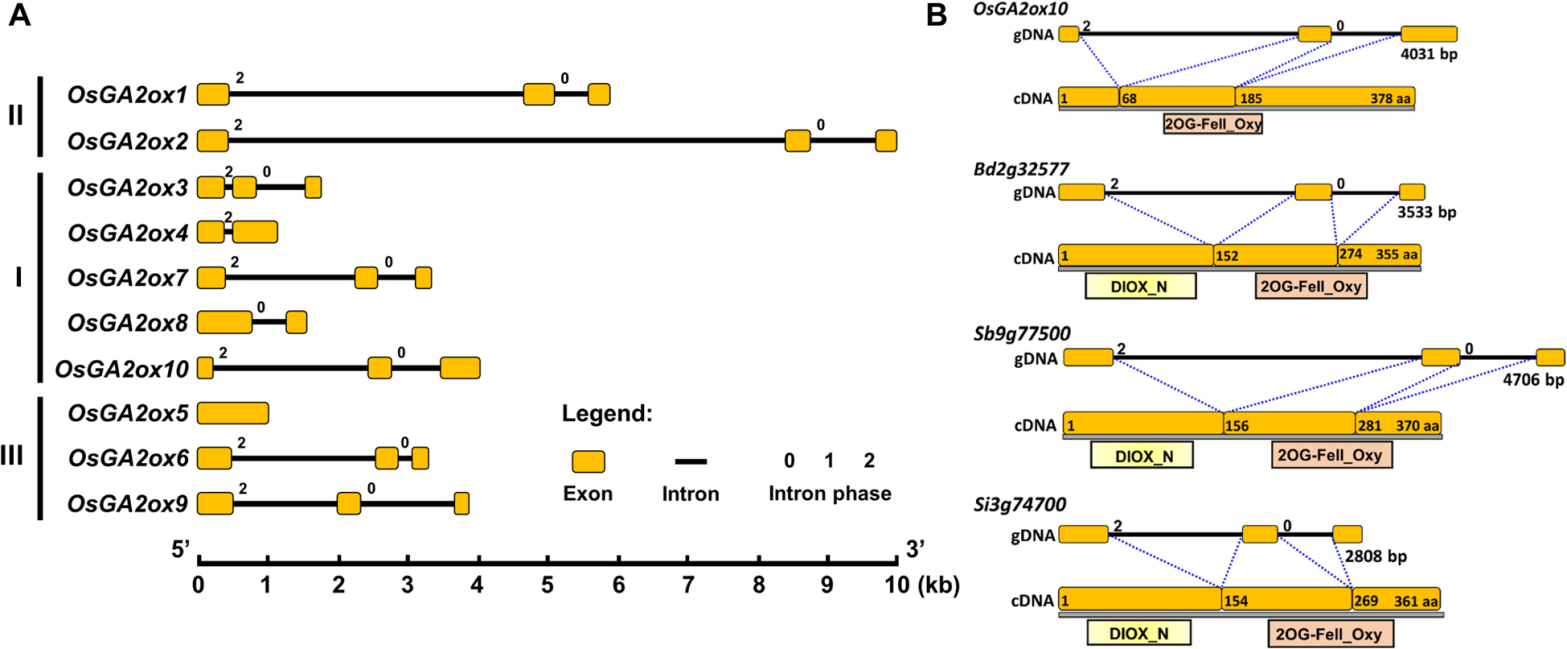
Gene structure of the rice GA2ox family members and comparison of the functional domains in *OsGA2ox10* and its orthologous genes. **a** Gene structures of all 10 rice GA2ox genes. The sizes of exons (box) and introns (line) are shown on the same scale. The numbers on top of each exon/intron junction indicate their intron phases. **b** Schematic diagram showing the exon-intron gene structure and amino acids encoded by the respective exons in *OsGA2ox10*, *Bd2g32577*, *Sb9g77500* and *Si3g74700*. The corresponding regions of the DIOX_N and 2OG-FeII_Oxy functional domains are presented below the exons, and DIOX_N and 2OG-FeII_Oxy are represented by yellow boxes and light pink boxes, respectively

The genomic structures of nine functionally confirmed (*OsGA2ox1 to 9*) and annotated (*OsGA2ox10*) genes were compared. The majority of OsGA2oxs contained three exons and two introns; however, *OsGA2ox4* and *OsGA2ox8* contained two exons, and *OsGA2ox5* contained a single exon (Fig. 9a). Based on sequence comparison, the large exon 2 of *OsGA2ox4* could have resulted from the fusion of exons 2 and 3; the large exon 1 of *OsGA2ox8* could have resulted from the fusion of exons 1 and 2; and the single exon of *OsGA2ox5* could have resulted from the fusion of the three exons. Other than the difference in numbers and lengths of the exons, significant intron length variations among them were also found. First, class II genes have the longest first intron range from 4 to 8 kb; second, class I OsGA2oxs could be divided into a relatively long first intron, such as that in *OsGA2ox7* and *OsGA2ox10*, and a very short first intron, such as that in *OsGA2ox3* and *OsGA2ox4*; and third, *OsGA2ox10* had longer first intron, a truncated first exon and an extended last exon (Fig. 9a).

In addition to genomic structure analysis, genomic sequence variation analysis of all *OsGA2oxs* among 4276 rice accessions (Zhao et al. 2015) was performed. Single-nucleotide polymorphisms (SNPs) and insertions/deletions (INDELs) in 5′-regulatory regions and INDELs and SNPs including nonsynonymous mutations (dNs) and synonymous mutations (dSs) in coding regions were collected from the RiceVarMap database (Zhao et al. 2015). The numbers of dN-, dS- and INDEL-causing frameshift mutations in the coding regions were calculated and compared (Table 2). The annotated *OsGA2ox10* gene contains 20 dNs, which was approximately 3-fold higher than the average number of dNs found in other OsGA2oxs, and *OsGA2ox10* has the highest number of INDEL and frameshift mutations, suggesting that the strength of selection constraints in the coding sequence of OsGA2ox10 was much less than that in other OsGA2ox genes. In addition, OsGA2ox10 contained no DIOX_N domain, which was different from the orthologs of other grass species that contained both DIOX_N and 2OG-FeII_Oxy conserved domains (Fig. 9b). In addition, transgenic rice overexpressing one *B. distachyon* ortholog of *OsGA2ox10*, *Bd2g32577*, which contains both conserved domains, showed a functional dwarf phenotype (Fig. 5b). With the evidence of high mutation rates in the coding region and abnormal gene structure, plus the nonexpression of *OsGA2ox10* observed in previous studies (Lo et al. 2008; Hirose et al. 2012), we inferred that *OsGA2ox10* is a pseudogene.

**Table 2:** Genomic sequence variations in the 5′-regulatory and coding regions of *OsGA2oxs* analyzed with 4276 rice accessions

Classes	Genes	5′-Regulatory region^a^	Coding region		
		SNP/INDEL^b^	dN	dS	FS/INDEL^c^
**I**	**OsGA2ox1**	**9/3**	**3**	**4**	**0/0**
	**OsGA2ox2**	**47/8**	**9**	**9**	**0/1**
**II**	**OsGA2ox3**	**23/7**	**4**	**4**	**0/0**
	**OsGA2ox4**	**48/22**	**10**	**5**	**1/1**
	**OsGA2ox7**	**37/1**	**7**	**5**	**0/2**
	**OsGA2ox8**	**45/9**	**3**	**0**	**0/0**
	**OsGA2ox10**	**29/5**	**20**	**12**	**3/7**
**III**	**OsGA2ox5**	**28/12**	**7**	**2**	**1/1**
	**OsGA2ox6**	**6/1**	**5**	**4**	**0/0**
	**OsGA2ox9**	**62/7**	**10**	**5**	**0/2**

^a^Sequences 1 kb upstream from the start codon were analyzed

^b^Numbers of SNPs and INDELs identified using/with the Rice/VarMap database (Zhao et al. 2015)

^c^Numbers of FSs (frameshift mutations) in coding regions caused by INDELs are shown on top of the total numbers of INDELs

## Discussion

### The Rice *GA2ox* Gene Family Comprises Nine Functional GA2oxs

Based on phylogenetic analysis, the existence of 10 rice *GA2ox* genes has been proposed, and out of these 10 genes, *OsGA2ox10* has been suggested to be a pseudogene (Lo et al. 2008). This is due to its irregular gene structure caused by the truncation of the first exon and extension of the last exon (Fig. 9a, b), and RNA expression of this gene is not detected in all analyzed tissues (Lo et al. 2008; Hirose et al. 2012). From genomic sequence variation analysis of *OsGA2oxs* among 4276 rice accessions, *OsGA2ox10* revealed the highest number of dN, INDEL and frameshift mutations in its coding region (Table 2), indicating that *OsGA2ox10* is the least stringent among OsGA2oxs under selective constraints. In addition, while transgenic rice overexpressing the ortholog *Bd2g32577* of *OsGA2ox10* from *B. distachyon*, which contains two typical conserved domains, showed a functional dwarf phenotype (Fig. 5b), no plant height-reducing effect was observed in the transgenic plants overexpressing synthetic *OsGA2ox10* cDNA (data not shown). We therefore conclude that *OsGA2ox10* is a pseudogene.

Other than the findings concerning *OsGA2ox10*, the present study demonstrated that five (*OsGA2ox1*, 2, 4, 7, and 8) additional rice GA2ox genes are functional in TNG67 based on the results of the T-DNA activation-tagged mutants (Fig. 1) and the ectopically overexpressed transgenic plants (Fig. 3). Although some T-DNA activation-tagged mutants such as M36548 for *OsGA2ox1* and M66925 for *OsGA2ox7* did not show a significant plant height reduction, the overexpression transgenic plants for each of these five genes did show significantly reduced height (Fig. 3). In addition, the RNA expression of the GA deficiency-inducible *OsGA20ox2* and *OsGA3ox2* genes was significantly induced in each of the *OsGA2ox* overexpression transgenic lines (Fig. 3). With the results of these five functional genes plus the previously identified four (*OsGA2ox3*, 5, 6, and 9) genes (Lo et al. 2008), we confirmed that the rice genome contains at least nine functional *GA2ox* genes.

### Members of the *OsGA2ox* Gene Family Exert Various Effects on Plant Height Reduction, Suggesting that their GA Deactivation Capability Diverged during Evolution

Although the plant height-reducing effects of the *OsGA2ox2*, *OsGA2ox4* and Os*GA2ox8* genes in T-DNA activation-tagged mutants (Fig. 1) had the same tendency as the effects from the overexpression transgenic approaches (Fig. 3), the reducing effects in the overexpression transgenic plants were much more severe than those in the activation-tagged mutants. This was because the plant height-reducing effects in the activation-tagged mutants can be influenced significantly by their T-DNA insertion events/locations (Liao et al. 2019) or by possible epigenomic changes around the T-DNA insertion sites (Jupe et al. 2019), as the mutant M36548 for *OsGA2ox1* showed activation of *OsGA2ox1* but caused little effect on plant height reduction, such that the GA deactivation capability could not be evaluated correctly solely on the basis of T-DNA activation-tagged mutants. In contrast, the GA deactivation capability of *OsGA2oxs* could be easily and correctly verified based on their plant height-reducing effects using the overexpression transgenic approach (Lo et al. 2017).

In the present study, transgenic plants overexpressing each of the *OsGA2ox* genes ectopically in the same setting were compared, and varying degrees of plant height reduction ranging from approximately 8% to 60% RPH were observed (Fig. 4a). Overexpressing each of the class II and class III genes caused dwarf phenotypes with approximately 12% to 31% RPH, and overexpressing each of the class I genes caused either severe dwarf (8% to 9% RPH for *OsGA2ox3*, *4*, and *8*) phenotypes or semidwarf (60% of RPH for *OsGA2ox7)* phenotypes (Fig. 4a). These various reducing effects on plant height based on the same comparison suggest that the capability of GA deactivation among rice GA2oxs could have diverged during evolution.

### *OsGA2ox2* can be Recognized as a Regulatory Hypofunctionalized Gene and is Functionally Preserved in the Rice Genome

Unlike the Rice Genome Annotation Project (http://rice.plantbiology.msu.edu/), which annotated *LOC_Os01g22920* as *OsGA2ox2* (Han and Zhu 2011; Liu et al. 2018), after experimental demonstration, we confirmed that the rice *GA2ox2* gene is the combination of *LOC_Os01g22920* and *LOC_Os01g22910*, which includes three exons constituting the typical DOXC class gene in 2OGD (Kawai et al. 2014) and a long first intron (Figs. 2c; 9a). In addition, our annotation for *OsGA2ox2* was identical to the annotation of the *Os01g0332200* gene from the RAP-DB database (https://rapdb.dna.affrc.go.jp/) (Kawahara et al. 2013; Sakai et al. 2013). Due to the low RNA expression level (Sakai et al. 2003) and large genomic DNA structure of *OsGA2ox2*, the function of this gene has not been characterized until now.

In the present study, we cloned the full-length cDNA from the T-DNA activation-tagged mutant M43852 and demonstrated that overexpression of *OsGA2ox2* has a plant height-reducing effect similar to that of *OsGA2ox1* (Figs. 3b, c; 4a). Since *OsGA2ox2* and *OsGA2ox1* are grouped into the same class II category (Additional file: Fig. S1), are located in the syntenic blocks between chromosomes #1 and #5 (Additional file: Fig. S6a, b), and thus have similar exon-intron structures and highly similar amino acid sequences, they were recognized as duplicated genes (Additional file: Figs. S1; S6b). However, their expression profiles were very different, as revealed by comparing the data collected from the Uniformed Viewer for Integrated Omics (UniVIO: http://univio.psc.riken.jp/; Additional file: Fig. S7a; Kudo et al. 2013) database, the Rice Expression Database (RED IC4R: http://ic4r.org; Additional file: Fig. S7b; Xia et al. 2017) and the Rice Expression Profile Database (RiceXPro; https://ricexpro.dna.affrc.go.jp/; Additional file: Fig. S7c; Sato et al. 2013). The RNA expression of *OsGA2ox2* was barely detectable in all analyzed tissues, and in contrast, the expression level of *OsGA2ox1* was significant in reproductive tissues, such as flowers (Additional file: Fig. S7a), panicles and anthers (Additional file: Fig. S7b, c).

**Fig. 10:**
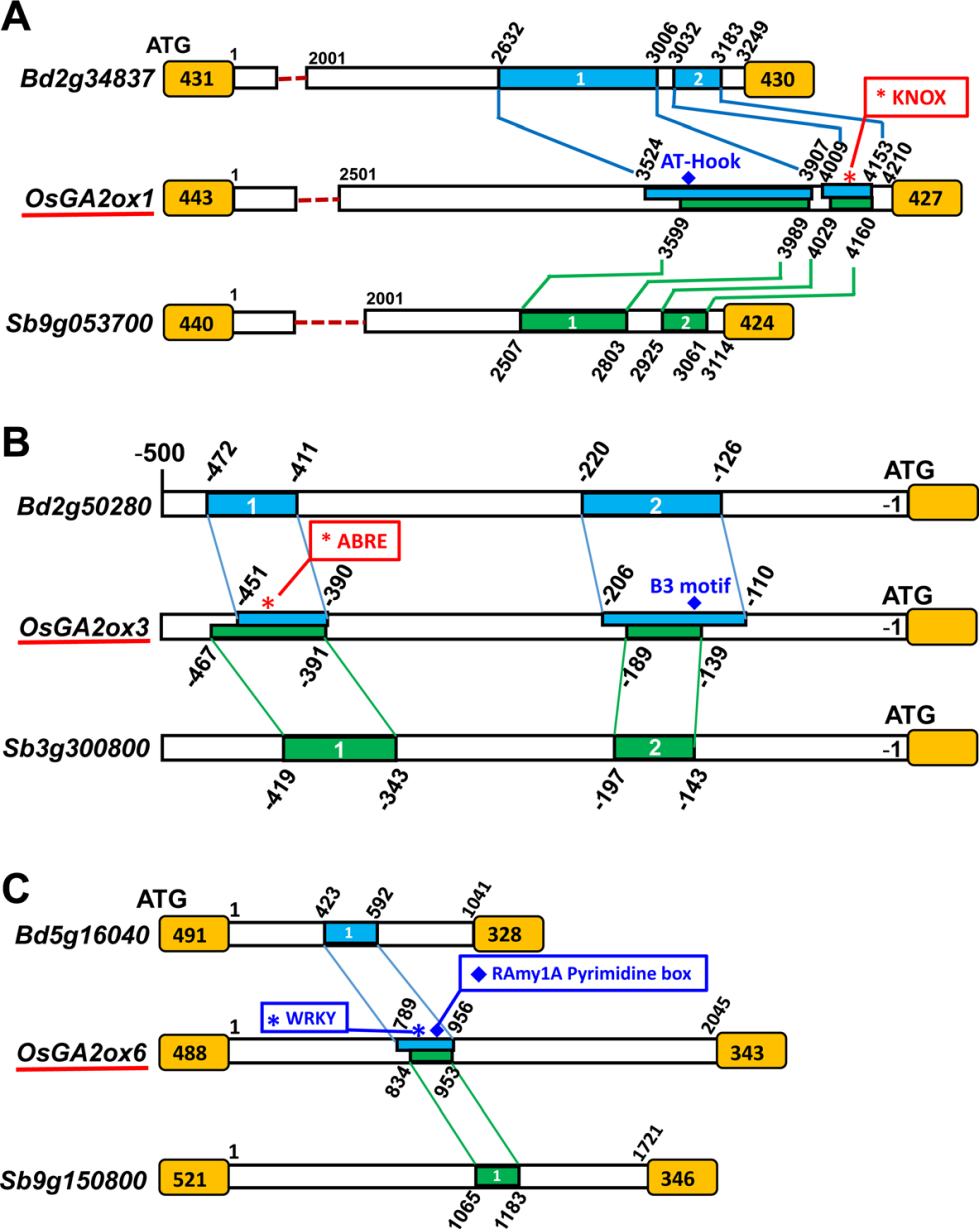
Conserved sequence motifs identified in the promoter and the first intron regions of rice *GA2ox1*, *GA2ox3*, *GA2ox6* and their orthologs in *B. distachyon* and *S. bicolor.* The sequences 1000 bp upstream of the start codon and the sequences of the first intron of all rice OsGA2oxs and their orthologs from *B. distachyon* and *S. bicolor* were analyzed by phylogenetic footprinting (http://plantpan.itps.ncku.edu.tw/index.html). The identified conserved regions are colored blue for *Bd* and green for *Sb*, and their relative sequence locations in each conserved region are shown. The exons are colored orange, and their sequence lengths are shown. The representative binding motifs with red boxes have been characterized, and those with blue boxes have not yet been characterized. **a** The relative locations of the AT-Hook motif in region 1 and the KNOX motif in region 2 of the first intron in *OsGA2ox1* are indicated. **b** The relative locations of the ABRE motif in region 1 and the B3 motif in region 2 of the *OsGA2ox3* promoter region are indicated. **c** The relative locations of the WRKY and RAmy1A Pyrmidine box motifs in the first intron of *OsGA2ox6* are indicated

Divergence in the expression between duplicated genes is prevalent in plants (Ganko et al. 2007; Li et al. 2009; Renny-Byfield et al. 2014) and might be caused by sequence variations in the cis-regulatory motifs between duplicated genes (Li et al. 2005). Phylogenetic footprinting analysis revealed that two regions in the first intron of *OsGA2ox1* were highly conserved across the Poaceae (Fig. 10a; Additional file: Table S1a), and a KN1 binding site that was bound by KNOX transcription factor to regulate *ZmGA2ox1* expression and that is responsible for the bioactive GA levels around the shoot apical meristem through KNOX protein regulation (Bolduc and Hake 2009) was included in the conserved region (Additional file: Table S2a). Other than the KNOX-binding site, many more conserved binding sites, such as AT-Hook and GATA binding sites, were also identified in these regions (Additional file: Table S2a). Although the exon-intron gene structure of *OsGA2ox2* was similar to that of *OsGA2ox1*, the first intron of *OsGA2ox2* was much longer than that of *OsGA2ox1* (Fig. 9a), and the conserved regulatory motifs in *OsGA2ox1* were absent in *OsGA2ox2* (Fig. 10a). In addition, the number of SNPs/INDELs in the 5′-regulatory region of *OsGA2ox2* was approximately 5 times higher than that of *OsGA2ox1* (Table 2). This evidence might explain why the expression differed between *OsGA2ox1* and *OsGA2ox2* (Additional file: Fig. S7). The phenomenon that OsGA2ox2 retained its GA deactivation capability (Fig. 4a) but decreased its overall expression levels was very similar to regulatory hypofunctionalization (Duarte et al. 2006). Therefore, *OsGA2ox2* could be recognized as a regulatory hypofunctionalized gene and a minor paralog in the class II OsGA2oxs.

From an evolutionary point of view, the expression difference that results from differences in regulatory motifs between duplicated genes is thought to be a critical way to preserve duplicated genes (Li et al. 2005; Ganko et al. 2007). The preservation of the duplicated gene *OsGA2ox2* in the rice genome might thus function as a genetic buffer against the detrimental effects caused by deleterious mutations (Duarte et al. 2006; Zhang 2012) or environmental stresses, as evidence has shown that, among 2OGD-encoding genes, *GA2ox* genes are particularly responsive to various abiotic stresses (Colebrook et al. 2014). Another evidence in multiple genes family, such as the three GID1a, GID1b1, and GID1b2 tomato GA receptors, showed that any single- and double-mutant plants were normal under optimal growth conditions, but a high degree of phenotypic variability was observed under changing environments, suggesting that all three genes are needed to maintain phenotypic stability under environmental stress (Illouz-Eliaz et al. 2019). We therefore could not rule out the possibility that the expression of *OsGA2ox2* might be induced under certain stress conditions to maintain phenotypic stability, as was the case for multiple *GID1* genes. However, future work is needed to explore this possibility.

### *OsGA2ox4* and *OsGA2ox8* can be Recognized as Regulatory Hypofunctionalized Genes in Clade A of Class I and are Functionally Preserved in the Rice Genome

Phylogenetic analysis of the rice *GA2ox* genes grouped *OsGA2ox3*, *4* and *8* into clade A of class I (Fig. 5a; Additional file: Fig. S1), and they are located in the syntenic blocks between chromosomes #1 and #5 (Additional file: Fig. S6b; *OsGA2ox1/OsGA2ox2* and *OsGA2ox7/OsGA2ox10* located in their corresponding syntenic blocks on chromosomes #1 and #5 were also observed), which was proposed to have occurred through a whole-genome duplication (WGD) event occurring approximately 96 million years ago before the Poaceae species divergence (Wang et al. 2015). Unlike the case of *OsGA2ox1* and *OsGA2ox2*, which have similar exon-intron gene structures, the second intron of *OsGA2ox4* and the first intron of *OsGA2ox8* were lost (Fig. 9a). This intron loss could be explained by either the RNA-based gene conversion model, the alternative-splicing-based model (Zhang et al. 2014) or other complex evolutionary mechanisms that were imposed on these two duplicated genes.

Although the exon-intron gene structures of *OsGA2ox4* and *OsGA2ox8* differ from the structure of *OsGA2ox3*, their proteins comprise similar amino acid sequences; moreover, when their full-length cDNAs were overexpressed in transgenic rice, the plants showed the same severe dwarf phenotype as that caused by *OsGA2ox3* (Figs. 3d, f; 4a). Even though the same enzyme activity assay performed for OsGA2ox3 (Sakai et al. 2003) and OsGA2ox4 (Liu et al. 2018) was not performed for OsGA2ox8, the GA deactivation capability reflected by plant height reduction was essentially the same among them (Fig. 4a). However, the expression profiles of *OsGA2ox4* and *OsGA2ox8* were very different from the profile of *OsGA2ox3* (Additional file: Fig. S7a, b). Ubiquitous expression of *OsGA2ox3* in various tissues has been reported (Sakai et al. 2003) and was further confirmed by at least two rice expression profile databases (Additional file: Fig. S7). By contrast, the expression of *OsGA2ox4* and *OsGA2ox8* was barely detectable in any tissue (Additional file: Fig. S7).

As mentioned above, expression differences between duplicated genes might be caused by sequence variations in the cis-regulatory motifs between duplicated genes (Li et al. 2005). Indeed, number of the sequence variations, counted by the numbers of SNPs/INDELs in the 5′-regulatory regions of *OsGA2ox4* and *OsGA2ox8*, was greater than the number in *OsGA2ox3* (Table 2). Additionally, phylogenetic footprinting analysis revealed two conserved regions in the 5′-regulatory region of *OsGA2ox3* and its orthologs from *B. distachyon* and *S. bicolor* (Fig. 10b), but these regions were absent in *OsGA2ox4* and *OsGA2ox8*. These conserved regions that contained several important regulatory motifs (Additional file: Table S2b) might be responsible for the expression differences among *OsGA2ox3*, *4* and *8.* While the expression of *OsGA2ox4* and *OsGA2ox8* was barely detectable in any tissue (Additional file: Fig. S7), the expression of *OsGA2ox4* in seedlings increased in response to irradiance to reduce the GA content (Hirose et al. 2012), and the expression of *OsGA2ox8* was reported to be regulated by the miR156-IPA1 regulatory mechanism in controlling seed dormancy (Miao et al. 2019). Overall, we proposed that these two duplicated genes could have evolved via regulatory hypofunctionalization and might function together as a genetic buffer against detrimental mutations, as mentioned above (Duarte et al. 2006; Zhang 2012).

### *OsGA2ox7* can be Recognized as a Functional Attenuated Gene and can be Used to Create Semidwarf Plants

Phylogenetic analysis in the Poaceae resulted in the division of class I GA2ox genes into clades A and B, and transgenic study demonstrated that the GA deactivation capability was very different between clade A and B genes (Fig. 5b, c). The *OsGA2ox7* clade B rice gene, in addition to its low expression levels in most tissues (Additional file: Fig. S7), showed much less GA deactivation capability than its paralogs *OsGA2ox3*, *OsGA2ox4*, *OsGA2ox8*; which draws interest to its functional role in rice and potential applications. It has been reported that the expression of *OsGA2ox7* was upregulated in rice seedlings in response to irradiance and was thought to be involved in seedling leaf sheath suppression under light (Hirose et al. 2012). However, the activation of *OsGA2ox7* in the T-DNA activation-tagged mutant M66925 showed a very minor effect on plant growth (Fig. 1d), suggesting that the majority of the bioactive GAs that decreased in rice seedlings under irradiance might not be due to elevated *OsGA2ox7* expression but instead is due to other elevated expression of other *GA2oxs*, such as *OsGA2ox4*, *OsGA2ox5*, *and OsGA2ox6* (Hirose et al. 2012). From an evolutionary point of view, we therefore propose that *OsGA2ox7* has hypofunctionally evolved in terms of both its transcription and GA deactivation capability and thus might have only a minor role in rice growth and development.

Even though OsGA2ox7 might not be crucially involved in rice growth and development, it may be a good candidate gene for overexpression and genetic engineering and thus improving the plant architecture, stress tolerance and possibly grain yield of rice, similar to the findings in our previous report involving point-mutated OsGA2ox6 variants (Lo et al. 2017). The semidwarfing trait causing approximately 55–60% RPH in nontransgenic (NT/WT) plants and an approximately 30% increase in tiller/panicle numbers (Table 1) makes *OsGA2ox7-OX* transgenic lines another useful option for future applications.

### GA Deactivation Capability Attenuated in Clade B GA2oxs is Associated with Four Amino Acid Variants

Two functionally distinct effects on growth suppression between clades A and B within class I were first observed and demonstrated in the present study (Figs. 4a; 5b, c). Thus, we proposed that certain amino acid variations or structural differences between these two clades exist to differentiate their GA deactivation capability. Indeed, the present study demonstrated at least four amino acid variants that are distinct between clade A (C186, C194, Q220 and Y274 in OsGA2ox3) and clade B (R191, P201, E227 and F283 in OsGA2ox7); these variants are associated with strong clade A (such as OsGA2ox3) and weak clade B (such as OsGA2ox7) GA deactivation capability.

Among these four amino acid variants, the functional importance of C186 and C194 in OsGA2ox3 was proposed via X-ray structural analysis and elucidated via an in vitro activity assay (Takehara et al. 2020). The crystal structure showed that the amino acid C194 was responsible for forming intermolecular disulfide bridges (C194–C194) and was crucial for tetramer formation, the *Km* for GA_4_ was reported as being approximately 10-fold lower for the tetramer with the monomer, while the *Vmax* was 4-fold higher for the tetramer (Takehara et al. 2020). Further sequence alignment showed that the amino acid C194 was conserved in clade A of class I (OsGA2ox3, 4 and 8) but diverged in clade B (OsGA2ox7 and 10) of class I and class II (OsGA2ox1 and 2) and was absent in class III (OsGA2ox5, 6 and 9). Another conserved amino acid, C186, in clade A but that varied in clade B was described as one of the three amino acids that form the hinge site of the entrance gate for substrate GA_4_s (Takehara et al. 2020), which might also be responsible for some enzymatic activity differences between clades A and B.

In the present study, overexpressing the OsGA2ox3 C194P variant (replacing C194 with Pro) in transgenic plants (*3P-OX*) resulted in minor effects on attenuating GA deactivation capability, increasing the RPH of NT from 13% to approximately 17% (Fig. 8c). By contrast, overexpressing the OsGA2ox3 C186R variant (replacing C186 with Arg) in transgenic plants (*3R-OX*) caused noticeable effects on attenuating the GA deactivation capability, increasing the RPH of NT from 13% to approximately 29% (Fig. 8c). Enhanced additive effects on attenuating GA deactivation capability that increased the RPH up to approximately 40% that of NT (Fig. 8c) were observed in transgenic plants (*3RP-OX*), in which overexpression of the C186R/C194P double variant (replacing C186 with Arg and C194 with Pro) demonstrated that both C186 and C194 were important for OsGA2ox3 activity *in planta*.

Two other amino acid variations between clades A (Q220 and Y274) and B (E227 and F283) did not involve the key amino acids at the catalytic pocket and did not display any possible interaction with GAs or cofactors in a previous report (Takehara et al. 2020), and *3E-OX* and *3F-OX* transgenic plants overexpressing their single-amino acid-replaced variants, showed no obvious effects on GA deactivation capability (Fig. 7). However, *3EF-OX* transgenic plants overexpressing the Q220E/Y274F double variant (replacing Q220 with Glu and Y274 with Phe) showed an obvious increase in RPH—up to approximately 24% that of NT (Fig. 7a), suggesting that amino acid sequences not involved in the formation of the catalytic pocket of OsGA2ox3 also affected its GA deactivation capability *in planta* and that a synergistic effect on these two conserved amino acids occurred. Although the Q220 and Y274 residues were outside the catalytic pocket (Additional file: Fig. S3), they were close to the iron (HTD) (Fig. 6b)- and 2OG-binding sites (RVS) (Fig. 6c), which might affect the interaction of OsGA2ox3 with iron and 2OG and thus affect the GA deactivation capability.

Overall, these four amino acids conserved in clade A were associated with their GA deactivation capability, and this capability could be attenuated when the conserved amino acids were replaced with the amino acids conserved in their corresponding site of clade B, suggesting that amino acid variations in clade B GA2oxs have evolved to attenuate their GA deactivation capability, such as that which occurred for OsGA2ox7. This evidence supports the different GA deactivation capabilities not only between OsGA2ox3 and OsGA2ox7 but also between clade A (*OsGA2ox4* and *OsGA2ox8* from rice; *Bd2g50280* and *Bd2g19900* from *B. distachyon*) and clade B (*Bd2g06670*, *Bd2g32577* from *B. distachyon*), which was demonstrated in the present study (Figs. 4a; 5).

### *OsGA2ox1*, *OsGA2ox3* and *OsGA2ox6* were Conserved and Dominant in their Respective Classes

Phylogenetic analysis based on the protein sequences classified GA2oxs into three classes (Lee and Zeevaart 2005), where the rice genes *OsGA2ox1* and *OsGA2ox2* belong to class II; *OsGA2ox3*, *OsGA2ox4*, *OsGA2ox7*, *OsGA2ox8*, and *OsGA2ox10* belong to class I; and *OsGA2ox5*, *OsGA2ox6*, and *OsGA2ox9* belong to class III (Additional file: Fig. S1). The cDNAs of nine (*OsGA2ox1* to *9*) *OsGA2ox* genes have been demonstrated to be functional and revealed various GA deactivation capabilities based on their effects on plant height reductions (Fig. 4a). The overexpression of *OsGA2ox1* in class II, the overexpression of *OsGA2ox3*, *OsGA2ox4*, and *OsGA2ox8* in class I and the overexpression of *OsGA2ox6* in class III revealed stronger GA deactivation capability than the others in their respective classes, which raises the question of whether any dominant paralogs exist in each class of the gene family. To explore this question, in addition to comparing their GA deactivation capability, we accounted for the exon-intron structures (Fig. 9), sequence differences in the promoter and coding regions (Table 2) and the expression profiles in various tissues (Additional file: Fig. S7) to understand the importance of each *OsGA2ox* gene in the family.

As the 5′-regulatory region and the intron region close to the transcription start site could be involved in regulating gene expression (Xie et al. 2018) by a mechanism called intron-mediated enhancement (IME) (Laxa 2016), the sequences 1000 bp upstream from the start codon and the sequences of the first intron of all rice *OsGA2oxs* and their orthologs from *B. distachyon* and *S. bicolor* were analyzed by phylogenetic footprinting (Chow et al. 2019). This analysis resulted in the identification of three genes, *OsGA2ox1*, *OsGA2ox3* and *OsGA2ox6*, containing conserved regions: two conserved regions (approximately 306–395 bp of region #1 and ~ 140–157 bp of region #2) in the first intron of *OsGA2ox1* (Fig. 10a), two conserved regions (~ 62–78 bp of region #1 and ~ 55–105 bp of region #2) in the promoter region of *OsGA2ox3* (Fig. 10b) and one conserved region (~ 120–170 bp) in the first intron of *OsGA2ox6* (Fig. 10c). The locations and lengths of these conserved regions (Additional file: Table S1) and their detailed binding motifs for transcription factors (TFs/motifs, Additional file: Table S2) are provided. Several conserved motifs, such as the KNOX (Bolduc and Hake 2009), AT-Hook (Matsushita et al. 2007) and GATA motifs (Richter et al. 2013) in the first intron of *OsGA2ox1* (Additional file: Table S2a), the ABRE/CE1 motifs (Cantoro et al. 2013) in *OsGA2ox3* (Additional file: Table S2b) and the NAC/NAM (Chen et al. 2015) and WRKY/Pyr motifs (Zhang et al. 2004) in OsGA2ox6 (Additional file: Table S2c), which are involved in GA-mediated growth and development regulations, were conserved across species within their respective classes of GA2ox genes.

For the *OsGA2ox1* and *OsGA2ox2* genes of class II, although their exon/cDNA sequences were similar, *OsGA2ox1* had less sequence variation in both the 5′-regulatory and coding regions than did *OsGA2ox2* (Table 2), which might explain why *OsGA2ox1* has a slightly better GA deactivation capability than *OsGA2ox2* does (Fig. 4a). In addition, the first intron of *OsGA2ox1* contains conserved regulatory motifs that were missing in the intron of *OsGA2ox2* (Figs. 9a; 10a), which might reduce *OsGA2ox2* RNA expression but maintain the expression of *OsGA2ox1* at a significant level in reproductive tissues (Sakamoto et al. 2001; Sakamoto et al. 2004) and at relatively high levels in other tissues (Additional file: Fig. S7). Moreover, a taller and approximately 5-day delay in heading date but with normal anthers and seed setting rate for the *osga2ox1* knockout mutant was observed in our preliminary results (Additional file: Fig. S8a), however no phenotypic difference was observed in the *osga2ox2* knockout mutant (data not shown). Overall, *OsGA2ox1* had stronger selection constraints, maintained higher RNA expression levels, and exhibited a better GA deactivation capability; thus, it could be recognized as the dominant paralog in class II.

For the class I genes, there are 5 members (when *OsGA2ox10* is included) of the family. Although *OsGA2ox4* and *OsGA2ox8* showed the same strong GA deactivation capability as *OsGA2ox3* did (Fig. 4a), their RNA expression levels were much lower than the level of *OsGA2ox3* (Additional file: Fig. S7). This differential expression might be explained by the evidence that *OsGA2ox3* has the least 5′-regulatory sequence variation among them, and the conserved 5′-regulatory motifs in *OsGA2ox3* were absent in all other members of the family (Fig. 10b). As mentioned above, *OsGA2ox7* can be recognized as a functional attenuated gene and *OsGA2ox10* was confirmed to be a pseudogene in the present study. Moreover, a recent report (Takehara et al. 2020) and our preliminary results using CRISPR/Cas9 to knock out the *OsGA2ox3* gene showed a taller phenotype (Additional file: Fig. S8b), while no phenotypic differences were observed among the *osga2ox4*, *osga2ox7*, and *osga2ox8* knockout mutants (data not shown). Overall, we propose that *OsGA2ox3* is the dominant paralog in class I.

*OsGA2ox5*, *OsGA2ox*6 and *OsGA2ox*9 are C_20_-type GA2oxs and were categorized into class III. Unlike *OsGA2ox5*, whose intronless gene structure (Fig. 9a) might have arose via a retroposition model (Han and Zhu 2011), *OsGA2ox6* and *OsGA2ox9* are phylogenetically conserved (Additional file: Fig. S1) and located in syntenic blocks on chromosomes #2 and #4 (Additional file: Fig. S6c), suggesting that *OsGA2ox6* and *OsGA2ox9* are duplicated genes that evolved through ancient WGD events (Han and Zhu 2011; Wang et al. 2015) before the Poaceae species divergence (Han and Zhu 2011; Wang et al. 2015). These three genes have been functionally characterized (Lo et al. 2008), and OsGA2ox6 displayed higher GA deactivation capability than OsGA2ox5 and OsGA2ox9 did (Fig. 4a).

Although no conserved region in the 5′-regulatory region was found, the 5′-regulatory sequences of *OsGA2ox6* were much more conserved than the paralogous *OsGA2ox5* and *OsGA2ox9* sequences were (Table 2), suggesting that *OsGA2ox6* was under stronger selective constraints, which might be important to maintain its transcriptional ability and specificity. Through phylogenetic footprinting assays, a conserved region in the first intron of *OsGA2ox6* (Fig. 10c) that contains transcriptional regulatory motifs, such as NAC/NAM- and WRKY/Pyr-binding motifs, was identified (Additional file: Table S2c). A NAC transcription factor, OsNAC2, is involved in suppressing the expression of GA biosynthesis-related genes and enhancing that of GA deactivation-related genes (Chen et al. 2015), and a WRKY transcription factor, OsWRKY71, functions as a transcriptional repressor to repress the expression of the GA-induced α-amylase gene *Amy32b* in aleurone cells (Zhang et al. 2004), suggesting that the conserved NAC/NAM- and WRKY/Pyr-binding motifs in *OsGA2ox6* might be important for *OsGA2ox6* transcriptional regulation. In addition, the *osga2ox6* mutant created by the CRISPR/Cas9 system presented chalky characteristics of its rice grains (Chen et al. 2019, and this study) and exhibited a taller phenotype in our preliminary study (Additional file: Fig. S8c), but the *osga2ox9* knockout mutant (Chen et al. 2019) and our *osga2ox5* knockout mutant (data not shown) did not show changes in plant height. Overall, we propose that *OsGA2ox6* is the dominant paralog in class III.

In summary, *OsGA2ox1* in class II, *OsGA2ox3* in class I and *OsGA2ox6* in class III had the least sequence variation in both the 5′-regulatory and coding regions (Table 2), contained conserved regulatory motifs in either 5′-regulatory or the first intron regions (Fig. 10), and revealed higher RNA expression levels (Additional file: Fig. S7) and stronger GA deactivation capability (Fig. 4a) than others did in their respective classes; thus, they can be recognized as the dominant paralogs in each of their respective classes, with preserved functional importance.

## Conclusions

In the present study we demonstrated that the rice GA2ox gene family contains nine functional genes and the class I GA2ox genes are divided into two functionally distinct clades in Poacea. Among them, the *OsGA2ox7* of clade B is a functional attenuated gene and that led to the identification of four conserved amino acids C186/C194 and *Q220/Y274* in clade A which were critically associated with GA deactivation capability. In addition, through sequence divergence, RNA expression profile and GA deactivation capability analyses, we proposed that *OsGA2ox1*, *OsGA2ox3* and *OsGA2ox6* are the three predominant paralogs in the family.

## Methods

### Plant Materials and Growth Conditions

The rice cultivar *Oryza sativa* Tainung 67 was used as the wild-type accession in this study, and the T-DNA mutants used in this study were obtained from the Taiwan Rice Insertional Mutants library (http://trim.sinica.edu.tw/). The seeds of wild-type plants and T-DNA mutants were surface sterilized with 2.5% sodium hypochlorite (NaClO) and then placed on MS media (Sigma-Aldrich, St. Louis, MO, USA) in a growth chamber at 28 °C under a 16 h/8 h light/dark cycle. Approximately 14–21 days later, the grown seedlings were transferred to a greenhouse or an isolated paddy field.

For the exogenous GA feeding experiment, the seeds from nontransgenic (NT) and OsGA2ox activation-tagged mutants or overexpression transgenic rice plants were grown on MS media for 7 days and then transferred to MS media consisting of 10 μM GA_3_; the shoot length of the seedlings was measured after 3 days of incubation.

*Brachypodium distachyon* seeds were incubated at 4 °C for 5–7 days. Then, the testa of seeds was removed, and seeds were placed on sterile water-soaked paper towels in petri dishes (90 × 15 mm), which were covered by tinfoil to avoid irradiance. After 2–3 days of incubation at 22 °C, the germinated seeds were transferred to pots in a growth chamber at 22 °C under a 20 h/4 h light/dark cycle.

### Plasmid Construction and Rice Transformation

For construction of GA2ox overexpression vectors, the full-length cDNA of GA2oxs from rice and *B. distachyon* were amplified with their respective cloning primer sets (Additional file: Table S3) together with Phusion High-Fidelity DNA Polymerase (Thermo Fisher Scientific, Waltham, MA, USA), after which the respective full-length GA2ox cDNA was further digested (by *SpeI* and *KpnI* digestion) and inserted downstream of the maize ubiquitin (Ubi) promoter in a pAHC18 vector (Bruce et al. 1989). For generation of point mutants in OsGA2ox3, site-directed mutagenesis was conducted as previously described (Lo et al. 2017), with minor modifications. The Ubi:OsGA2ox3 vector was used as a template and amplified by point-mutation primer sets (Additional file: Table S3) in conjunction with Phusion High-Fidelity DNA Polymerase, and the Ubi:OsGA2ox3 template in the PCR product was removed by *DpnI* digestion. Then, point mutants of the Ubi:OsGA2ox3 vector were purified by an Illustra GFX PCR DNA and Gel Band Purification Kit (GE Healthcare, Chicago, IL, USA). The respective Ubi:GA2ox overexpression vectors were linearized by *HindIII* digestion and then inserted into a pCAM1301 binary vector (Hajdukiewicz et al. 1994) to form transformation vectors. For construction of CRISPR/Cas9 expression vectors, the potential sgRNAs that target each of the *OsGA2ox* genes were designed using E-CRISPR (Heigwer et al. 2014) (http://www.e-crisp.org/E-CRISP/) and these respective pair of sgRNAs (Additional file: Table S3) were synthesized, annealed and integrated into the pRGEB32 vector (Xie et al. 2015) driven by the OsU3 promoter. The resulting plasmid vectors were further transformed into *Agrobacterium tumefaciens* strain EHA-105 and used for rice transformation as previously described (Hiei et al. 1997). At least 10 transgenic T_0_ lines for overexpression constructs and 3 to 5 transgenic lines for CRISPR knockout mutants were obtained and analyzed. Ten or more transgenic lines were used to measure their plant heights and at least three lines were used for RNA expression analysis. For phenotypic comparisons, one or three representative plants were shown. For analysis of CRISPR knockout mutants, the target site sequence modifications were first analyzed at T_0_ generation, then confirmed at the next generation and using at least 12 progenies from T_1_ or the following generations to cross out their CRISPR/Cas9 constructs.

### Southern Blotting and T-DNA Insertion Site Identification

For Southern blotting assays, 15 μg of genomic DNA extracted with CTAB extraction buffer (Doyle and Doyle 1987) was digested by restriction enzymes and subjected to 1% (w/v) agarose gel electrophoresis. The fractionated DNA was then transferred to an Amersham Hybond-N^+^ membrane (GE Healthcare, Chicago, IL, USA) and hybridized with a P^32^-labeled GUS DNA probe. P^32^-labeled GUS DNA probes were prepared by an Amersham Rediprime II DNA labeling system (GE Healthcare, Chicago, IL, USA) according to the manufacturer’s instructions.

For T-DNA insertion identification, the inverse PCR method was conducted as previously described, with minor modifications (Kim et al. 2011), where 25 μg of genomic DNA was digested by *SacI* or *EcoRI* in a 100 μL volume for 8 h. Afterward, the digested DNA fragments were purified by the phenol-chloroform extraction method (Sambrook and Russell 2006) and subjected to 100 μL of ligation mixture consisting of 15 units of T4 DNA ligase (Promega, Madison, WI, USA), after which the ligation reaction was incubated at 4 °C for 48 h. The ligation products were further purified by the phenol-chloroform extraction method and quantified to 500 ng/μL for inverse PCR, which was conducted by using the LB2-B/Hpt-R and RB/GUS-R primer sets (Additional file: Table S3).

### RNA Extraction and Gene Expression Analysis

For target OsGA2ox gene expression analysis in the T-DNA mutants, since most OsGA2oxs were not expressed in leaves after the transition to the reproductive stage (Lo et al. 2008), flag leaves from wild-type plants and T-DNA mutants at the ripening stage were collected for RNA extraction to investigate whether targeted OsGA2oxs were activated in T-DNA mutants. The shoots of 15-day-old *B. distachyon* seedlings were used for RNA extraction for *Bd2g50280*, *Bd2g19900, Bd2g06670* and *Bd2g32577* gene cloning.

Total RNA was extracted by using TRIzol reagent (Invitrogen, Carlsbad, CA, USA), and RNase-free DNase I (Thermo Fisher Scientific, Waltham, MA, USA) was used to remove possible DNA contaminants from the RNA samples. Five μg of DNA-free RNA was used in cDNA synthesis by a RevertAid First-Strand cDNA Synthesis Kit (Thermo Fisher Scientific, Waltham, MA, USA) according to the recommended protocol of the manufacturer in a 20 μL reaction volume. One μL of synthesized cDNA and 0.6 units of GoTaq DNA polymerase (Promega, Madison, WI, USA) were used for PCR for gene expression analysis by the respective gene-specific primer sets (Additional file: Table S3) in a 15 μL reaction volume.

The expression sequence data of the respective OsGA2oxs in other rice cultivars were downloaded from UniVIO (Kudo et al. 2013) (http://univio.psc.riken.jp/), the Rice Expression Database (RED) (Xia et al. 2017) (http://expression.ic4r.org/) and RiceXPro (Sato et al. 2013) (https://ricexpro.dna.affrc.go.jp/index.html) and then analyzed, and LOC_*Os01g22910* was used as the representative transcript of OsGA2ox2 in these expression data.

### Phylogenetic Analysis and SNP Calculation

In the sequence similarity search, the protein sequence of OsGA2ox7 was used as a query to identify homologs in basal angiosperms (*Amborella trichopoda*), basal monocots (*S. polyrhiza*, *Z. marina*) and Poaceae (*B. distachyon*, *O. sativa*, *S. bicolor*, *S. italica*) by the BLASTP program, with an E-value threshold of 10^− 5^; the protein sequences of the investigated species were downloaded from the Phytozome v12 database (https://phytozome.jgi.doe.gov/pz/portal.html#). The 2OGD protein conserved domain analysis was determined by using Pfam (El-Gebali et al. 2019), as typical GA2oxes have two Pfam domains: DIOX_N (PF14226) and 2OG-FeII_Oxy (PF03171). Protein sequences from the start of DIOX_N to the end of 2OG-FeII_Oxy were aligned by MAFFT version 7 (Katoh and Standley 2013) with the L-INS-I model, and the resulting multiple sequence alignment data were trimmed by trimAl v1.2, with a cutoff value of 0.6 (all columns were removed when the gap percentage surpassed 40%). Then, the sequences were removed from the alignment if more than 40% gaps were present. The trimmed alignment data were used in phylogenetic tree construction by RAxML version 8 (Stamatakis 2014) with the parameters –m PROTGAMMAJTT and a bootstrap analysis with 400 replicates.

The sequence variation of the 5′-regulatory region (1 kb upstream of the start codon) and coding region among the respective OsGA2oxs were retrieved from Rice Variation Map (RiceVarMap) version 2.0 (Zhao et al. 2015). The number of SNPs/INDELs in the 5′-regulatory region was a combination of both single-nucleotide polymorphisms (SNPs) and insertions/deletions (INDELs) within the region, and the numbers of nonsynonymous (dN), synonymous (dS) or frameshift mutations in the coding region were calculated based on the information within RiceVarMap. For phylogenetic footprinting analysis, the sequences of the 5′-regulatory region (1 kb upstream of the start codon) and intron region of the respective GA2oxs in *O. sativa*, *B. distachyon* and *S. bicolor* were aligned by the “cross species” function of Plant Promoter Analysis Navigator (PlantPan) version 3.0 (http://plantpan.itps.ncku.edu.tw/index.html) (Chow et al. 2019), with an E-value threshold of 10^− 5^. The exon-intron gene structure of the OsGA2oxs was displayed by Gene Structure Display Server (GSDS) version 2.0 (Hu et al. 2015). The characteristic motifs among class I GA2oxs were identified by Multiple Em for Motif Elicitation (MEME) software, and the parameters were set as previously described (Bailey et al. 2015; Huang et al. 2015).

### Statistical Analysis

All data were analyzed with Statistical Product and Service Solutions (International Business Machines Corporation, Armonk, New York, USA). For multiple comparisons, the Turkey’s honestly significant difference (HSD) test was used. Different letters represent differences at a significance level of *P*-value < 0.05. For comparison with the control, the Student’s *t*-test was used, and significance levels are indicated as: * = 0.01 < *P* < 0.05, ** = 0.001 < *P* < 0.01, *** = *P* < 0.001. The comparisons between the gene-edited plants and wild-type (WT) were presented by means of boxplots.

### Accession Numbers and Gene Loci

The following gene loci can be found in the Phytozome database or RAP-DB:

**Table:** 

OsActin: LOC_Os03g61970	OsGA2ox1: LOC_Os05g06670
OsGA2ox2: Os01g0332200 (RAP-DB)	OsGA2ox3: LOC_Os01g55240
OsGA2ox4: LOC_Os05g43880	OsGA2ox5: LOC_Os07g01340
OsGA2ox6: LOC_Os04g44150	OsGA2ox7: LOC_Os01g11150
OsGA2ox8: LOC_Os05g48700	OsGA2ox9: LOC_Os02g41954
OsGA2ox10: LOC_Os05g11810	OsGA20ox2: LOC_Os01g66100
OsGA3ox2: LOC_Os01g08220	Bd2g50280: Bradi2g50280
Bd2g19900: Bradi2g19900	Bd2g06670: Bradi2g06670
Bd2g16727: Bradi2g16727	Bd2g16750: Bradi2g16750
Bd2g32577: Bradi2g32577	Sb3g300800: Sobic.003G300800
Sb9g196300: Sobic.009G196300	Sb3g022700: Sobic.003G022700
Sb9g230800: Sobic.009G230800	Sb9g077500: Sobic009G077500
Si5g323400: Seita.5G323400	Si5g147400: Seita.5G147400
Si3g148100: Seita.3G148100	Si5g266500: Seita.5G266500
Si3g074700: Seita.3G074700	

## Supplementary Information

**Additional file 1 **: **Figure S1**. Phylogenetic tree based on comparisons of OsGA2ox amino acid sequences. **Figure S2**. Effects of exogenous GA_3_ treatment on seedling growth. **Figure S3**. Relative positions of amino acids Q220 and Y274 in OsGA2ox3 according to the resolved 3-D protein structure. **Figure S4**. Phenotypic comparison of WT-OX, 3E-OX, 3F-OX and 3EF-OX transgenic rice plants and their RNA expression analysis. **Figure S5**. Phenotypic comparison of WT-OX, 3R-OX, 3P-OX and 3RP-OX transgenic rice plants and their RNA expression analysis. **Figure S6**. Locations of and evolutionary relationships among the *OsGA2ox* genes on rice chromosomes. **Figure S7**. Expression analysis of *OsGA2ox* genes in various tissues according to data collected from different rice expression databases. **Figure S8**. Phenotypic comparisons of CRISPR/Cas9 knockout *OsGA2ox1*, *OsGA2ox3*, and *OsGA2ox6* mutants. **Table S1A**. The locations and aligned lengths of the two conserved regions in the first intron of *OsGA2ox1* and its orthologs in *Bd* and *Sb*. **Table S1B**. The locations and aligned lengths of the conserved regions in the 5′-regulatory region of *OsGA2ox3* and its orthologs in *Bd* and *Sb*. **Table S1C**. The locations and aligned lengths of the conserved region in the first intron of *OsGA2ox6* and its orthologs in *Bd* and *Sb*. **Table S2A**. DNA-binding motifs in the first intron region of OsGA2ox1. **Table S2B**. DNA-binding motifs in promoter regions of OsGA2ox3. **Table S2C**. DNA-binding motifs in the first intron region of OsGA2ox6. **Table S3**. List of primers and their sequences used in this study.

## Data Availability

Not applicable.
