# Supplementary material for: Comparisons within the Rice GA 2-Oxidase Gene Family Revealed Three Dominant Paralogs and a Functional Attenuated Gene that Led to the Identification of Four Amino Acid Variants Associated with GA Deactivation Capability
Source: Rice (N Y). 2021 Jul 28;14:70. doi: 10.1186/s12284-021-00499-4 (PMC8319247; doi:10.1186/s12284-021-00499-4)
Supplement: Supplementary file 1 — Additional file 1 : Figure S1. Phylogenetic tree based on comparisons of OsGA2ox amino acid sequences. Figure S2. Effects of exogenous GA3 treatment on seedling growth. Figure S3. Relative positions of amino acids Q220 and Y274 in OsGA2ox3 according to the resolved 3-D protein structure. Figure S4. Phenotypic comparison of WT-OX, 3E-OX, 3F-OX and 3EF-OX transgenic rice plants and their RNA expression analysis. Figure S5. Phenotypic comparison of WT-OX, 3R-OX, 3P-OX and 3RP-OX transgenic rice plants and their RNA expression analysis. Figure S6. Locations of and evolutionary relationships among the OsGA2ox genes on rice chromosomes. Figure S7. Expression analysis of OsGA2ox genes in various tissues according to data collected from different rice expression databases. Figure S8. Phenotypic comparisons of CRISPR/Cas9 knockout OsGA2ox1, OsGA2ox3, and OsGA2ox6 mutants. Table S1A. The locations and aligned lengths of the two conserved regions in the first intron of OsGA2ox1 and its orthologs in Bd and Sb. Table S1B. The locations and aligned lengths of the conserved regions in the 5′-regulatory region of OsGA2ox3 and its orthologs in Bd and Sb. Table S1C. The locations and aligned lengths of the conserved region in the first intron of OsGA2ox6 and its orthologs in Bd and Sb. Table S2A. DNA-binding motifs in the first intron region of OsGA2ox1. Table S2B. DNA-binding motifs in promoter regions of OsGA2ox3. Table S2C. DNA-binding motifs in the first intron region of OsGA2ox6. Table S3. List of primers and their sequences used in this study. [file 12284_2021_499_MOESM1_ESM.docx]

**Supplementary Data**

**Running title: GA deactivation capabilities of rice GA 2-oxidase genes**

**Comparisons within the Rice GA 2-Oxidase Gene Family Revealed Three Dominant Paralogs and a Functional Attenuated Gene that Led to the Identification of Four Amino Acid Variants Associated with GA Deactivation Capability**

***Corresponding author:**

L-J Chen,

Institute of Molecular Biology, National Chung Hsing University, Taichung 40227, Taiwan

Tel: +886-4-22851885

E-mail address: ljchen@nchu.edu.tw

Subject areas:

1) growth and development,

3) regulation of gene expression,

10) genomics, evolution,

Number of color figures: 10

Number of tables: 2

Number of supplementary figures: 8

Number of supplementary tables: 3

**Supplementary figures**

**
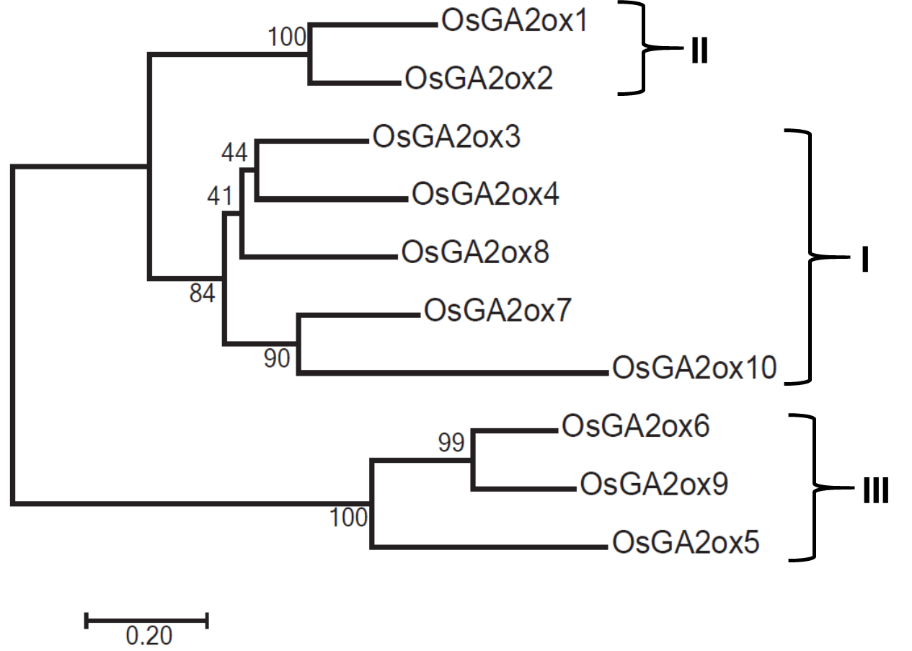
**

**Figure S1. Phylogenetic tree based on comparisons of OsGA2ox amino acid sequences**

The OsGA2ox protein sequences were aligned by MAFFT version 7, and the resulting alignment was used to generate a phylogenetic tree that was constructed by the neighbor-joining method using bootstrap analysis with 400 replicates.


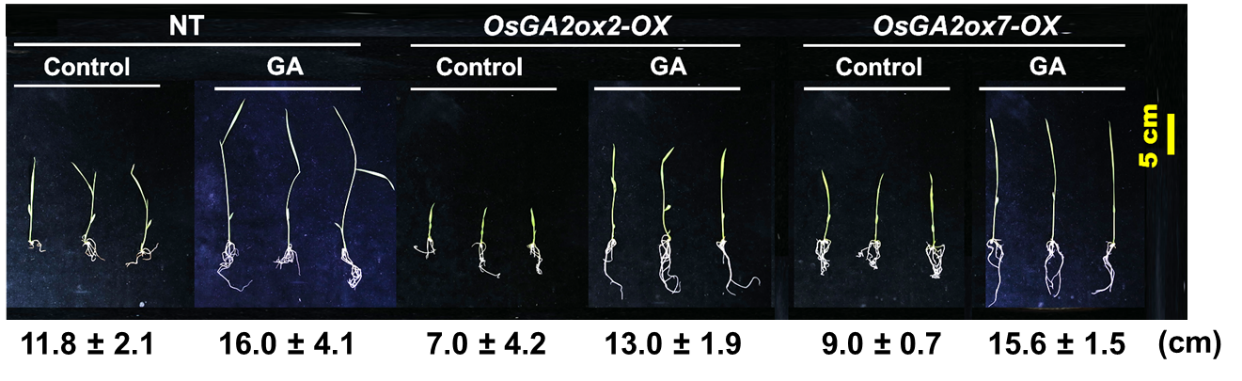


**Figure S2. Effects of exogenous GA_3_ treatment on seedling growth**

Seeds from nontransgenic (NT), *OsGA2ox2-OX* and *OsGA2ox7-OX* rice plants were grown on MS media for 7 days and then transferred to MS media supplemented with 10 µM GA_3_ (GA) or no GA (Control) for 3 days to measure their shoot length. The average shoot lengths are shown below the picture. *OsGA2ox2-OX* and *OsGA2ox7-OX* could be rescued by exogenous GA_3_ treatment.

**
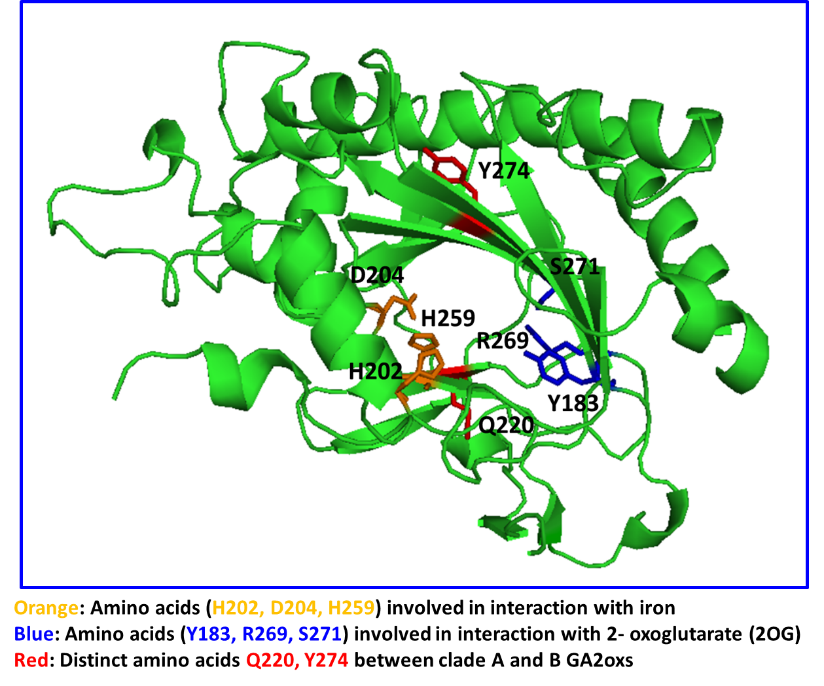
**

**Figure S3. Relative positions of amino acids Q220 and Y274 in OsGA2ox3 according to the resolved 3-D protein structure**

The protein structure was downloaded from the Protein Data Bank under accession number 6KU3, the amino acids involved in interaction with iron are shown in orange, the amino acids involved in interaction with 2-oxoglutarate (2OG) are shown in blue, and Q220 and Y274 are shown in red.


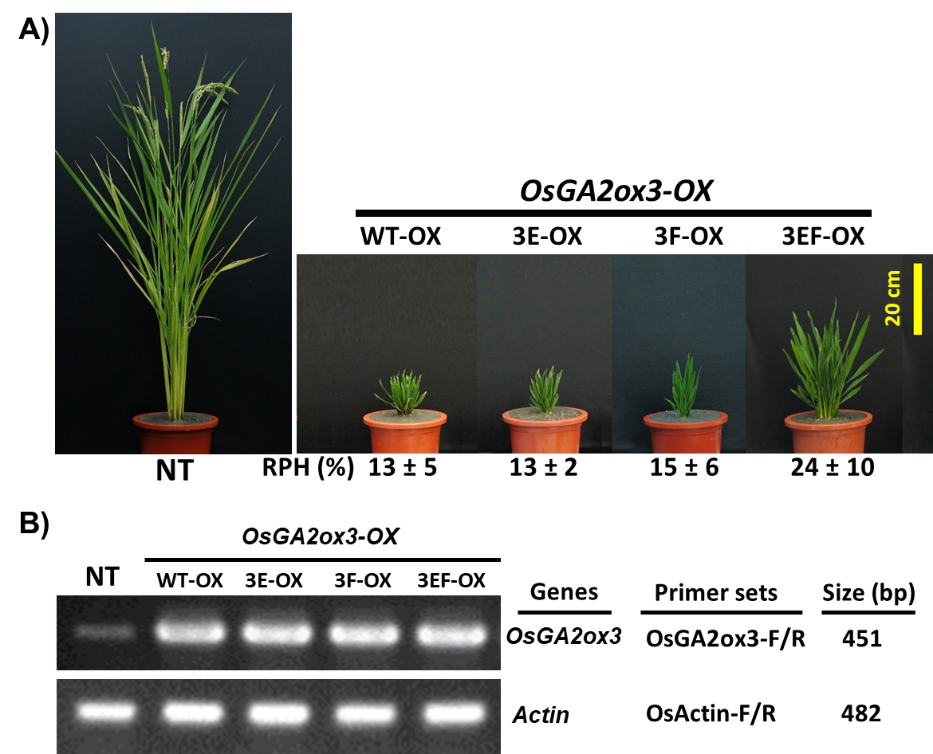


**Figure S4. Phenotypic comparison of WT-OX, 3E-OX, 3F-OX and 3EF-OX transgenic rice plants and their RNA expression analysis**

1. Phenotypic comparison of WT-OX, 3E-OX, 3F-OX and 3EF-OX transgenic rice plants and their nontransgenic (NT) plants. A representative transgenic plant with the average RPH resulting from each overexpression line is shown. Values are means ± SE (*n* = 5).
2. Results of RT-PCR analysis of *OsGA2ox3* gene in WT-OX, 3E-OX, 3F-OX and 3EF-OX transgenic rice plants and NT plant. The Actin gene was used as control.


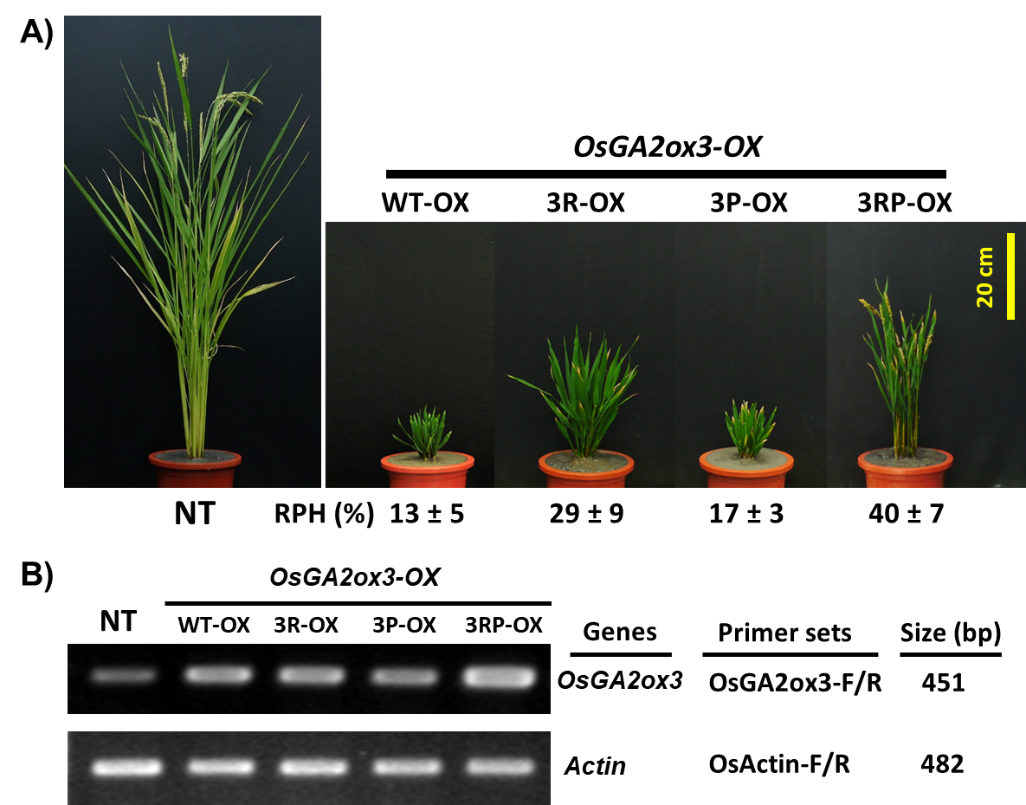


**Figure S5. Phenotypic comparison of WT-OX, 3R-OX, 3P-OX and 3RP-OX transgenic rice plants and their RNA expression analysis**

**(A)** Phenotypic comparison of WT-OX, 3R-OX, 3P-OX and 3RP-OX transgenic rice plants. A representative overexpression transgenic plant with the average RPH from each overexpression line is shown. Values are means ± SE (*n* = 5).

(B) Results of RT-PCR analysis of *OsGA2ox3* gene in WT-OX, 3R-OX, 3P-OX and 3RP-OX transgenic rice plants and NT plant. The Actin gene was used as control.


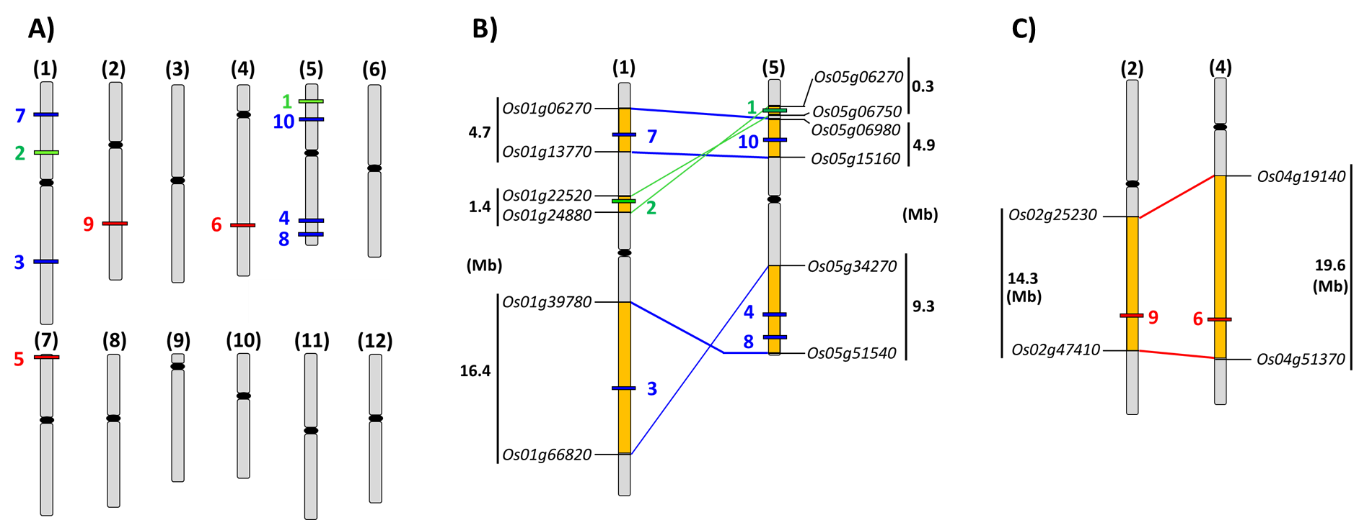


**Figure S6. Locations of and evolutionary relationships among the *OsGA2ox* genes on rice chromosomes**

1. The relative locations of the 10 *OsGA2ox* family genes on rice chromosomes.
2. Schematic representation of syntenic blocks between chromosomes #1 and #5 and the relative locations of each C_19_-type OsGA2ox in the syntenic regions are indicated.
3. Schematic representation of syntenic blocks between chromosomes #2 and #4 and the locations of OsGA2ox6 and OsGA2ox9 in the syntenic regions are indicated.

The locus IDs from the Rice Genome Annotation Project were used to define the boundary of the syntenic blocks. The syntenic blocks defined by Thiel’s study (Thiel et al. 2009) are shown as colored boxes. The chromosome locations of each *OsGA2ox* gene, boundary loci and syntenic blocks were visualized using the Oryzabase Map tool (Kurata and Yamazaki 2006) (<http://viewer.shigen.info/oryzavw/maptool/MapTool.do>).

Kurata N, Yamazaki Y (2006) Oryzabase. An integrated biological and genome information database for rice. *Plant Physiol* 140: 12-17

Thiel T, Graner A, Waugh R, Grosse I, Close TJ, Stein N (2009) Evidence and evolutionary analysis of ancient whole-genome duplication in barley predating the divergence from rice. *BMC Evol Biol* 9: 209


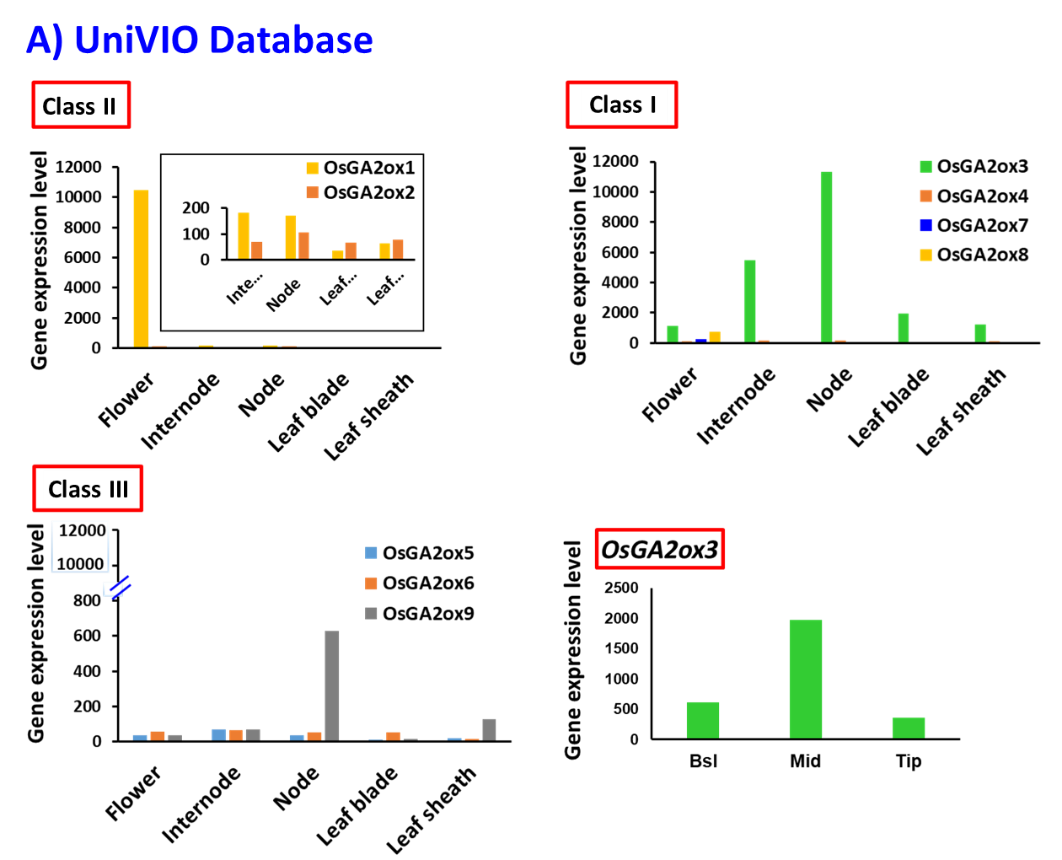


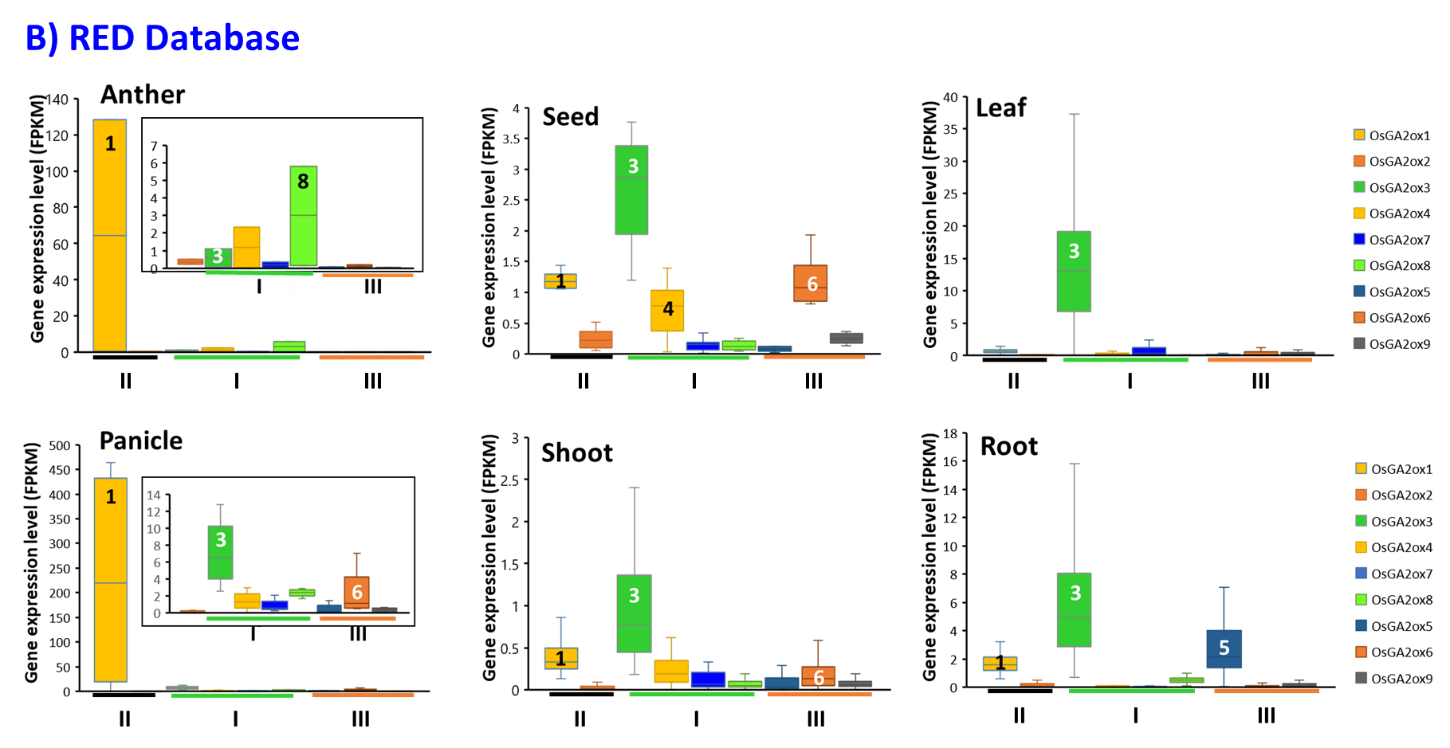


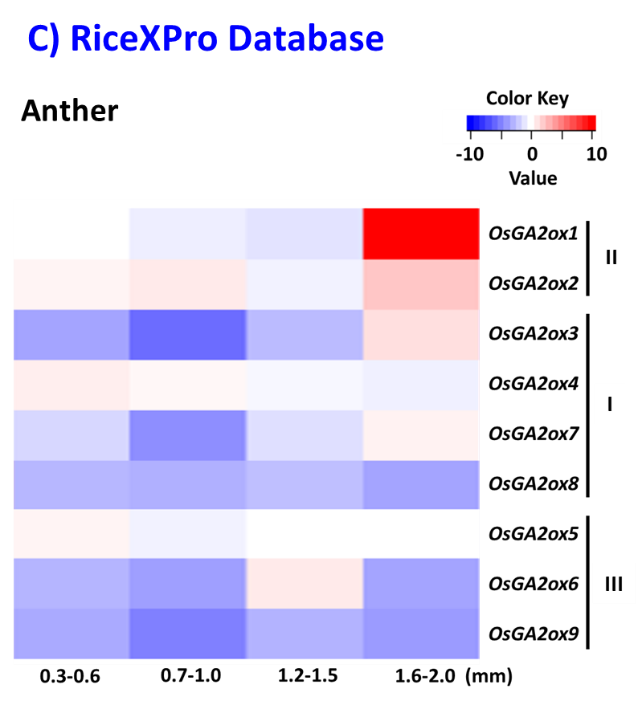


**Figure S7. Expression analysis of *OsGA2ox* genes in various tissues according to data collected from different rice expression databases**

1. Expression profiles of *OsGA2oxs* in various rice tissues represented by different GA2ox classes; the data were obtained from the UniVIO (<http://univio.psc.riken.jp/>) database. The “Bsl”, “Mid” and “Tip” stand for the “basal”, “middle” and “tip” part of the flag leaf blade respectively.
2. Expression profiles of *OsGA2oxs* in various tissues that classified by different tissues; the data were obtained from the RED database (<http://expression.ic4r.org/>).
3. Expression heat map of *OsGA2oxs* in anthers at different developmental stages; the data were obtained from the RiceXPro database (<https://ricexpro.dna.affrc.go.jp/>).

***
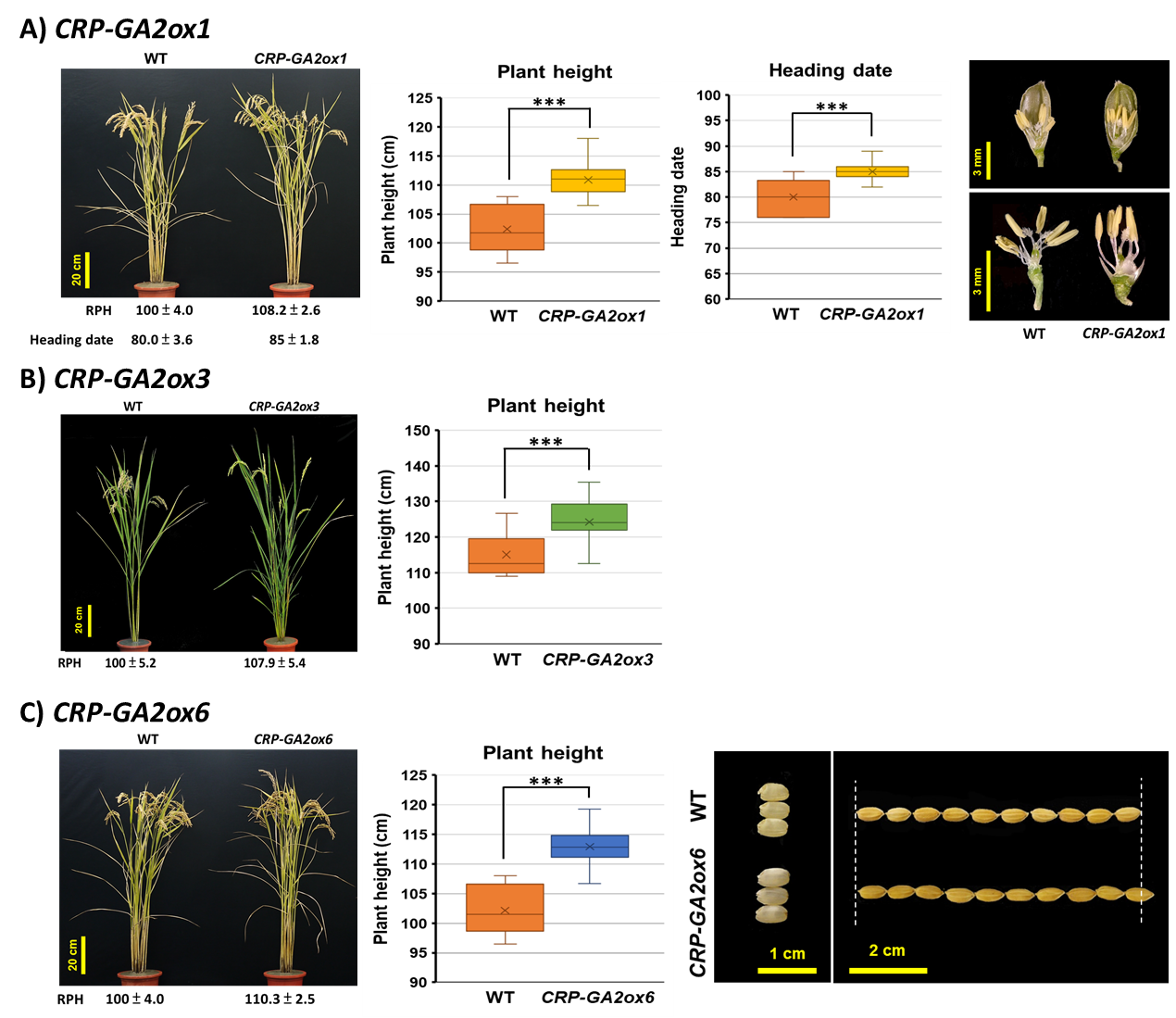
***

**Figure S8. Phenotypic comparisons of CRISPR/Cas9 knockout *OsGA2ox1*, *OsGA2ox3*, and *OsGA2ox6* mutants**

Knockout mutants of *OsGA2ox1*, *OsGA2ox3* and *OsGA2ox6* were created via CRISPR/Cas9 editing technology. At least two independent transformation events and two or three editing patterns that resulted in knocking out the function of the genes were obtained. Quantitative data were obtained from edited plants that were determined to be T-DNA free and that remained homozygous-edited for at least two seasons. The relative plant height (RPH %) means ± SE were shown for each of the representative mutant plants and the absolute plant height (cm) from at least 10 samples were analyzed and presented by boxplots. The statistical comparisons using Student’s *t*-test was to show the differences between the mutants and the wild-type (WT) control and their significance levels were determined as **P* < 0.05, ***P* < 0.01, ****P* < 0.001. Representative knockout mutants of *osga2ox1* **(A, *CRP-GA2ox1*),** *osga2ox3* **(B, *CRP-GA2ox3*)**, and *osga2ox6* **(C, *CRP-GA2ox6*),** and statistical analysis of plant heights for *CRP-GA2ox1, CRP-GA2ox3, CRP-GA2ox6* and heading date for *CRP-GA2ox1* are shown. The phenotype comparisons of anthers for *CRP-GA2ox1* and the bigger and chalky rice grains for *CRP-GA2ox6* are shown.

**Table S1A. The locations and aligned lengths of the two conserved regions in the first intron of *OsGA2ox1* and its orthologs in *Bd* and *Sb***

| **Intron conserved region #1** | | | | |  | **Intron conserved region #2** | | | |
| --- | --- | --- | --- | --- | --- | --- | --- | --- | --- |
| **Gene** | **Location** | **Identity** | **Alignment length** | **E-value** |  | **Location** | **Identity** | **Alignment length** | **E-value** |
| *Bd2g34837* | 2632 to 3006 | 80.8 | 395 | 1E-91 |  | 3032 to 3183 | 73.9 | 157 | 1E-15 |
| ***OsGA2ox1*** | 3524 to 3907 |  |  |  |  | 4009 to 4153 |  |  |  |
|  | 3599 to 3989 | 82.7 | 306 | 9E-80 |  | 4029 to 4160 | 74.3 | 140 | 2E-17 |
| *Sb9g053700* | 2507 to 2803 |  |  |  |  | 2925 to 3061 |  |  |  |

**Table S1B. The locations and aligned lengths of the conserved regions in the 5’-regulatory region of *OsGA2ox3* and its orthologs in *Bd* and *Sb***

| **5’-regulatory conserved region #1** | | | | |  | **5’-regulatory conserved region #2** | | | |
| --- | --- | --- | --- | --- | --- | --- | --- | --- | --- |
| **Gene** | **Location** | **Identity** | **Alignment length** | **E-value** |  | **Location** | **Identity** | **Alignment length** | **E-value** |
| *Bd2g50280* | -472 to -411 | 85.5 | 62 | 3E-16 |  | -220 to -126 | 71.4 | 105 | 3E-9 |
| ***OsGA2ox3*** | -451 to -390 |  |  |  |  | -206 to -110 |  |  |  |
|  | -467 to -391 | 79.5 | 78 | 1E-13 |  | -189 to -139 | 87.3 | 55 | 3E-15 |
| *Sb3g300800* | -419 to -343 |  |  |  |  | -197 to -143 |  |  |  |

**Table S1C. The locations and aligned lengths of the conserved region in the first intron of *OsGA2ox6* and its orthologs in *Bd* and *Sb***

| **Intron conserved region #1** | | | | |
| --- | --- | --- | --- | --- |
| **Gene** | **Location** | **Identity** | **Alignment length** | **E-value** |
| *Bd5g16040* | 423 to 592 | 83.5 | 170 | 7E-47 |
| ***OsGA2ox6*** | 789 to 956 |  |  |  |
|  | 834 to 953 | 89.2 | 120 | 1E-40 |
| *Sb9g150800* | 1065 to 1183 |  |  |  |

**Table S2A. DNA-binding motifs in the first intron region of OsGA2ox1**

| **TF/Motif Name** | **Location** | **Sequence** | **Matrix ID/Literature** |
| --- | --- | --- | --- |
| **OsGA2ox1 CR1 (3599~3898)** | | | |
| α-Amylase | 3606~3611 | AATAAA | TF_motif_seq_0282 |
| RAmy3D TATCCAY motif | 3644~3650 | TATCCAT | TF_motif_seq_0387 |
|  | 3890~3896 | TATCCAT |  |
| ANAERO1 | 3659~3665 | AAACAAA | TF_motif_seq_0343 |
| AT-Hook | 3603~3611 | GAGAATAAA | TFmatrixID_0131 |
|  | 3793~3801 | TGGAAAAAA | TFmatrixID_0133 |
|  | 3827~3836 | ATTTTTGCCC |  |
| B3 | 3680~3684 | CATGC | TF_motif_seq_0256 |
|  | 3863~3867 | GCATG |  |
|  | 3894~3898 | CATGC |  |
| GATA | 3611~3619 | AATGATCCA | TFmatrixID_0262 |
|  | 3756~3764 | TGTGATCCA |  |
| GT1 | 3706~3711 | GATAAA | TF_motif_seq_0321 |
|  | 3795~3800 | GAAAAA |  |
|  | 3840~3846 | GGAAAAT |  |
| Homeodomain; TALE | 3812~3821 | TCCTGTCAAC | TFmatrixID_0293 |
|  | 3848~3852 | TGTCA | TF_motif_seq_0246 |
| NF-YB | 3808~3812 | CCAAT | TF_motif_seq_0257 |
|  | 3837~3841 | ATTGG |  |
| TBP | 3649~3655 | ATATAAA | TFmatrixID_0418 |
| TCP | 3620~3624 | GGCCC | TF_motif_seq_0266 |
| **OsGA2ox1 CR2 (4029~4153)** | | | |
| AT-Hook | 4096~4104 | TTTTTACTT | TFmatrixID_0133 |
| GT1 | 4149~4154 | GATAAA | TF_motif_seq_0321 |
| Homeodomain; TALE | 4063~4067 | AGTCA | TF_motif_seq_0246 |
|  | 4104~4108 | TGTCA |  |
| Homeodomain; WOX | 4057~4064 | TCAATCAG | TFmatrixID_0299 |
| Homeodomain; HD-ZIP | 4055~4066 | CATCAATCAGTC | TFmatrixID_0295 |
| NAC; NAM | 4062~4070 | CAGTCAAAG | TFmatrixID_0382 |
| WRKY | 4061~4071 | TCAGTCAAAGA | TFmatrixID_0451 |
| KNOX | 4054~4069 | CCATCAATCAGTCAAA | Bolduc and Hake (2009) |

**Table S2B. DNA-binding motifs in promoter regions of OsGA2ox3**

| **TF/Motif Name** | **Location** | **Sequence** | **Matrix ID/Literature** |
| --- | --- | --- | --- |
| **OsGA2ox3 CR1 (-451~-391)** | | | |
| RAmy3 CGACG element | -431~-427 | CGTCG | TF_motif_seq_0259 |
|  | -415~-411 | CGACG |  |
| TCP | -425~-421 | GGCCC | TF_motif_seq_0266 |
| ABRE | -433~-428 | CACGTC | Cantoro (2013) |
| CE1 | -394~-390 | CACCA |  |
| **OsGA2ox3 CR2 (-189~-139)** | | | |
| B3 | -149~-145 | CATGC | TF_motif_seq_0256 |
| Storekeeper | -174~-166 | CCCGACCTC | TFmatrixID_0417 |

**Table S2C. DNA-binding motifs in the first intron region of OsGA2ox6**

| **TF/Motif Name** | **Location** | **Sequence** | **Matrix ID/Literature** |
| --- | --- | --- | --- |
| **OsGA2ox6 CR1 (834~953)** | | | |
| ANAERO3 | 844~850 | GTGATGA | TF_motif_seq_0390 |
| AP2 | 948~957 | AACGCCGTCC | TFmatrixID_0103 |
| B3 | 885~889 | CATGC | TF_motif_seq_0256 |
| C2H2 | 898~910 | TAGTACACTAACC | TFmatrixID_0214 |
| Homeodomain; TALE | 835~839 | AGTCA | TF_motif_seq_0246 |
| NAC; NAM | 834~842 | GAGTCAATG | TFmatrixID_0382 |
| RAmy1A Pyrimidine box | 893~898 | CCTTTT | TF_motif_seq_0309 |
| WRKY | 834~841 | GAGTCAAT | TFmatrixID_0445 |

**Table S3. List of primers and their sequences used in this study**

| **Name of primer sets** | **Sequence** | **Size of PCR product** | |  |
| --- | --- | --- | --- | --- |
| **For gene cloning** |  |  | |  |
| OsGA2ox1-SpeI-F | 5’-TT**ACTAGT**ATGGTGGTGCCTTCCGCGACGA-3’ | 1166 bp | |  |
| OsGA2ox1-KpnI-R | 5’-AGA**GGTACC**CTATGCTTTTCCCTCACTGGCAT-3’ |  |  |  |
| OsGA2ox2-SpeI-F | 5’-AAG**ACTAGT**ATGGTGGTGCCGGCTGCTG-3’ | 1195 bp | |  |
| OsGA2ox2-KpnI-R (3'-UTR) | 5’-AG**GGTACC**CAACAAACTTCCAGCGAT-3’ |  |  |  |
| OsGA2ox3-SpeI-F | 5’-AGA**ACTAGT**ATGGTGGTTCTCGCTGGC-3’ | 1002 bp | |  |
| OsGA2ox3-KpnI-R | 5’-GAT**GGTACC**CTACTTCTTCTCAAACTGGGC-3’ |  |  |  |
| OsGA2ox4-SpeI-F | 5’-GAA**ACTAGT**ATGGTGGTGCTCGCGAA-3’ | 1083 bp | |  |
| OsGA2ox4-KpnI-R | 5’-GCT**GGTACC**TTAGGCGAGTGGGTTAGCG-3’ |  |  |  |
| OsGA2ox7-SpeI-F | 5’-GAA**ACTAGT**ATGGTGGTGCTTGCCAAGG-3’ | 1026 bp | |  |
| OsGA2ox7-KpnI-R | 5’-CCT**GGTACC**CTAGTTCTCAAATCTGCAGAGCC-3’ |  |  |  |
| OsGA2ox8-SpeI-F | 5’-GAA**ACTAGT**ATGGTGGCGATCACGGCG-3’ | 1080 bp | |  |
| OsGA2ox8-KpnI-R | 5’-CCT**GGTACC**TTATTTCTTCGTCGCGGC-3’ |  |  |  |
| Bd2g50280-SpeI-F | 5’-AGA**ACTAGT**ATGGTGGTTCTCGCCAGC-3’ | 999 bp | |  |
| Bd2g50280-KpnI-R | 5’-GAT**GGTACC**GGTACCCTACTTCTGAAACTGGGC-3’ |  |  |  |
| Bd2g19900-SpeI-F | 5’-GAA**ACTAGT**ATGGTGGTCCTGGCGAAGC-3’ | 1083 bp | |  |
| Bd2g19900-KpnI-R | 5’-GCT**GGTACC**TTAGGACCGGTGGTGGAGGC-3’ |  |  |  |
| Bd2g06670-SpeI-F | 5’-GAA**ACTAGT**ATGGTGGTCCTTGCCAAGGG-3’ | 1095 bp | |  |
| Bd2g06670-KpnI-R | 5’-CCT**GGTACC**CTAGTTCTCGAAGAGGCAGAGC-3’ |  |  |  |
| Bd2g32577-SpeI-F | 5’-GAA**ACTAGT**ATGGTGGTTCTTGCCCAGGGCG-3’ | 1097 bp | |  |
| Bd2g32577-KpnI-R | 5’-CCT**GGTACC**CAATGGCGTGCCTAGCTGGTGGTC-3’ |  |  |  |
| **For point mutations** |  |  | |  |
| OsGA2ox3-Q220E-F | 5’-GGCACGTCCGGCCTGGAGATCGCGCTCCGCGAC-3’ | | 5960 bp | |
| OsGA2ox3-Q220E-R | 5’-GTCGCGGAGCGCGATCTCCAGGCCGGACGTGCC-3’ | |  |  |
| OsGA2ox3-Y274F-F | 5’-CTAGGGTTTCCTTCATCTTCTTTGGAGGGCCACCG-3’ | |  |  |
| OsGA2ox3-Y274-R | 5’-CGGTGGCCCTCCAAAGAAGATGAAGGAAACCCTAG-3’ | |  |  |
| OsGA2ox3-C186R-F | 5’-GAACCACTACCCGCCGCGCCGCGCGCTGCAGG-3’ | |  |  |
| OsGA2ox3-C186R-R | 5’-CCTGCAGCGCGCGGCGCGGCGGGTAGTGGTTC-3’ | |  |  |
| OsGA2ox3-C194P-F | 5’-CTGCAGGGGCTCGGCCCGAGCGTCACCGGCTTC-3’ | |  |  |
| OsGA2ox3-C194P-R | 5’-GAAGCCGGTGACGCTCGGGCCGAGCCCCTGCAG-3’ | |  |  |

**Table S3. List of primers and their sequences used in this study (continued)**

| **Name of primer sets** | **Sequence** | **Size of PCR product** |
| --- | --- | --- |
| **For genotyping, inverse PCR and Southern blotting** | |  |
| M36548-F | 5’-TTGTGTGCTTAATAGGTTGATGC-3’ | 765 bp |
| M36548-R | 5’-CTCTTCATTTCATTCTATCTCCGT-3’ |  |
| M43852-F | 5’-GGCTTCTCCTACACCAACACATC-3’ | 1480 bp |
| M43852-R | 5’-CACACCTTCTTGACGCGCTC-3’ |  |
| M96803-F | 5’-CCTGCTCTTGGTACAAGGGAC-3’ | 1287 bp |
| M96803-R | 5’-CATAGGCAGCAGAAATATGTGAC-3’ |  |
| M66925-F | 5’-CCACCAACAGAGAAGCCAAGACG-3’ | 956 bp |
| M66925-R | 5’-GGACAAGCCGACGCGCATC-3’ |  |
| M61685-F | 5’-CTATTGGGCTGCTACCATGGC-3’ | 1902 bp |
| M61685-R | 5’-ACTCCCAGGCTACAGGCCAT-3’ |  |
| 2ox7_L1-F | 5’-CCAAGAAGTCCAAGCAAGCC-3’ | 758 bp |
| 2ox7_L1-R | 5’-CATACCAAGCATCAGCATAGGAAA-3’ |  |
| 2ox7_L3-F | 5’-CGGCGTGTATGTGTTTCCTT-3’ | 552 bp |
| 2ox7-L3-R | 5’-GAGAGGAATGGCTGGCGAT-3’ |  |
| Hpt-F | 5’-GCTTCGATGTAGGAGGGCGTG-3’ | 542 bp |
| Hpt-R | 5’-GCTCCAGTCAATGACCGCTGTTAT-3’ |  |
| GUS-F | 5’-ACGTCCTGTAGAAACCCCA-3’ | 413 bp |
| GUS-R | 5’-AGTTCAGTTCGTTGTTCACACA-3’ |  |
| RB | 5’-AACTCATGGCGATCTCTTACC-3’ |  |
| LB2-B | 5’-GTGAGCAGTTCCCAGATAAGG-3’ |  |
| **For OsGA2ox CRISPR knockout** | |  |
| OsGA2ox1_sgRNA-F | 5’-**GGCA**GGTGGCGAGGCAGGTGGCGA-3’ |  |
| OsGA2ox1_sgRNA-R | 5’-**AAAC**TCGCCACCTGCCTCGCCACC-3’ |  |
| OsGA2ox3_sgRNA-F | 5’-**GGCA**GCGCGCTGGTGACGGCGGAA-3’ |  |
| OsGA2ox3_sgRNA-R | 5’-**AAAC**TTCCGCCGTCACCAGCGCGC-3’ |  |
| OsGA2ox6_sgRNA-F | 5’-**GGCA**GTACCCGGCGTGCCCTTTCG-3’ |  |
| OsGA2ox6_sgRNA-R | 5’-**AAAC**CGAAAGGGCACGCCGGGTAC-3’ |  |

**Table S3. List of primers and their sequences used in this study (continued)**

| **Name of primer sets** | **Sequence** | **Size of PCR product** |
| --- | --- | --- |
| **For RT-PCR** |  |  |
| OsGA2ox1-F | 5’-CGAGCAAACGATGTGGAAGGGCTACAGG-3’ | 332 bp |
| OsGA2ox1-R | 5’-TGGCTCAGGCGGAGTGAGTACATTGTCG-3’ |  |
| OsGA2ox2-F (LOC_Os01g22910-F) | 5’-CCCCACATCCCTGACAAGGCTC-3’ | 592 bp |
| OsGA2ox2-R (LOC_Os01g22910-R) | 5’-CTATTCATGGTCGTCATCGTCC-3’ |  |
| LOC_Os01g22920-F | 5’-GGCGTTCTTCGTGCGGGC-3’ | 292 bp |
| LOC_Os01g22920-R | 5’-CGAGTTCATATCTTTGTCAAGTTGCTG-3’ |  |
| OsGA2ox3-F | 5’-TGAGCGCGCTGGTGACGGCGGA-3’ | 451 bp |
| OsGA2ox3-R | 5’-CTTGATTTGTAGGCAGCCTTC-3’ |  |
| OsGA2ox4-F | 5’-TCGGTGGAGGATAACTTCGGC-3’ | 999 bp |
| OsGA2ox4-R | 5’-TGGGTTAGCGACAGGTGGTGG-3’ |  |
| OsGA2ox7-F | 5’-ACGGGAGCTTCTACGCGAGT-3’ | 594 bp |
| OsGA2ox7-R | 5’-TCAAATCTGCAGAGCCTGTCGTC-3’ |  |
| OsGA2ox8-F | 5’-GTGCTGCGGCGGATGGTGGTGG-3’ | 555 bp |
| OsGA2ox8-R | 5’-CTTCGTCGCGGCCTCATCGTTGG-3’ |  |
| OsGA20ox2-F | 5’-TACTACAGGGAGTTCTTCGCGGACAGCA-3’ | 268 bp |
| OsGA20ox2-R | 5’-TGTGCAGGCAGCTCTTATACCTCCCGTT-3’ |  |
| OsGA3ox2-F | 5’-TCTCCAAGCTCATGTGGTCCGAGGGCTA-3’ | 346 bp |
| OsGA3ox2-R | 5’-TGGAGCACGAAGGTGAAGAAGCCCGAGT-3’ |  |
| OsActin-F | 5’-CTTGTATGTGACAATGGCACTG-3’ | 482 bp |
| OsActin-R | 5’-CCTTCATAGATTGGCACTG-3’ |  |
| Bd2g50280-F | 5’-TGCTCCGCTCCAACGGCACCTC-3’ | 161 bp |
| Bd2g50280-R | 5’-CACCACCCTATGCTTCACGCTCTT-3’ |  |
| Bd2g19900-F | 5’-CCGCAGCTCGTCTCCATCCTTCA-3’ | 337 bp |
| Bd2g19900-R | 5’-CCGCCTTCTTGTACTCGCCC-3’ |  |
| Bd2g06670-F | 5’-AGATCATCTCCGTGCTCCGCTC-3’ | 185 bp |
| Bd2g06670-R | 5’-CTCGCTGTTCACCACCACCCTGT-3’ |  |
| Bd2g32577-F | 5’-GCTGGAGCTGATGGCGGAAG-3’ | 481 bp |
| Bd2g32577-R | 5’-CGTGAACTCCCTGTAGCGGC-3’ |  |
